# MFAP2 secreted by TGF-β1-induced cancer-associated fibroblast cells promotes gastric cancer peritoneal metastasis through Src-STAT3-PTK7 axis

**DOI:** 10.1038/s41420-026-03243-3

**Published:** 2026-07-08

**Authors:** Yuen Tan, Shulan Sun, Wentao Wang, Yujian Zhang, Zexing Shan, Fan Sun, Yan Zhao, Huimian Xu, Yao Xing, Jianjun Zhang

**Affiliations:** 1 Department of Gastric Surgery, Cancer Hospital of Dalian University of Technology (Liaoning Cancer Hospital & Institute), Shenyang, Liaoning China; 2 The Liaoning Provincial Key Laboratory of Interdisciplinary Research on Gastrointestinal Tumor Combining Medicine with Engineering, Shenyang, Liaoning China; 3 Central Laboratory, Cancer Hospital of Dalian University of Technology (Liaoning Cancer Hospital & Institute), Shenyang, Liaoning China; 4 Department of Surgical Oncology, First Affiliated Hospital of China Medical University, Shenyang, Liaoning China

**Keywords:** Gastric cancer, Integrin signalling

## Abstract

Peritoneal metastasis is the leading risk factor for gastric cancer (GC). However, the mechanism of gastric cancer peritoneal metastasis (GCPM) is still unclear. GCPM depends not only on the “seeds” of tumor cells but also on the “soil” of the peritoneal microenvironment. This study aimed to analyze the changes in the peritoneal tissue microenvironment of nine patients with early-stage GC, advanced-stage GC, and GCPM using single-cell transcriptome sequencing. It found that the number of microfiber-associated protein 2 (MFAP2)-positive cancer-associated fibroblasts (CAFs) gradually increased with tumor progression and was associated with poor prognosis of GC during GCPM progression. Mechanistically, GC cells secreted transforming growth factor-β1 (TGF-β1) to stimulate the entry of Smad4 into the nucleus. This promoted the transcription of MFAP2 in peritoneal mesothelial cells and led to mesothelial-mesenchymal transition (MMT) of peritoneal mesothelial cells into CAFs, thereby altering the peritoneal microenvironment and facilitating the colonization of GC cells on the peritoneum. Moreover, MFAP2 secreted by CAFs bound to the integrin αVβ3 receptor on the surface of GC cells, activating the Src-STAT3 signaling pathway and upregulating the expression of protein tyrosine kinase 7 (PTK7) in GC cells. PTK7 accumulated intracellular β-catenin and facilitated its nuclear entry, activating transcription of downstream target genes to enhance the invasion and adhesion of GC cells, thereby promoting GCPM progression. Our findings provide insights into how changes in the peritoneal microenvironment promote the peritoneal metastasis of GC, thus providing new molecular targets for personalized treatment and prognostic evaluation in clinical practice.

**Figure:**
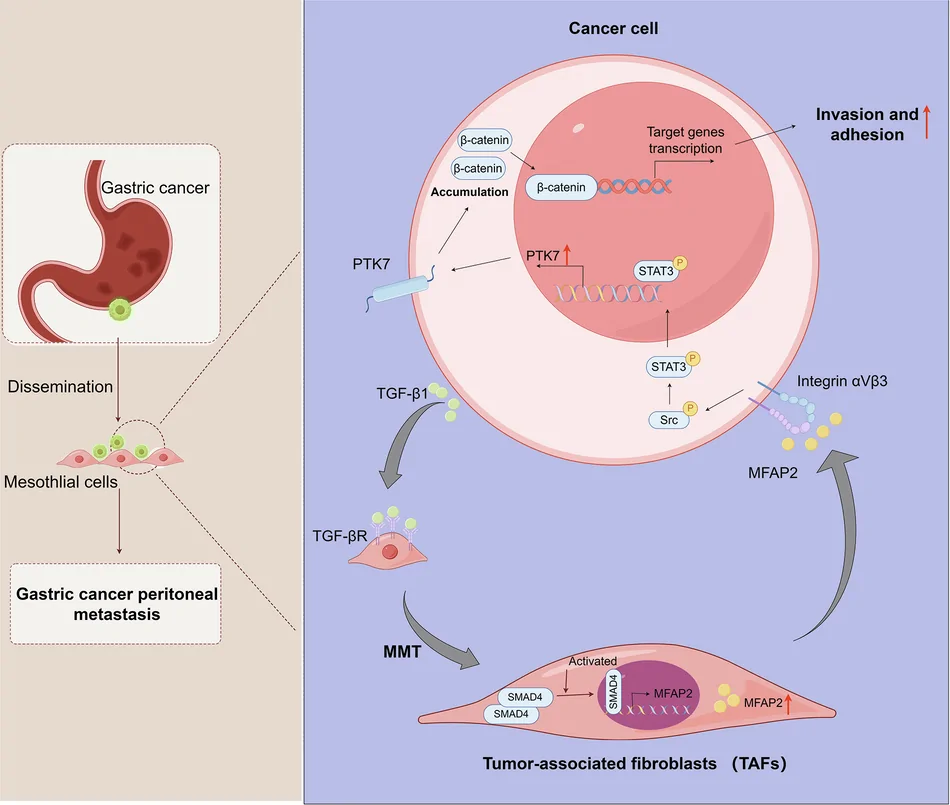

## Introduction

Peritoneal metastasis is the most common form of metastasis in advanced gastric cancer (GC) and the leading cause of mortality among patients [1]. Compared with patients with non-gastric cancer peritoneal metastasis (GCPM), patients with GCPM have a poor prognosis, with a median survival time of only 3-6 months, which is accompanied by clinical manifestations such as refractory ascites and abdominal wall nodules [2, 3]. Compared with patients with non-gastric cancer peritoneal metastasis (GCPM), patients with GCPM have a poor prognosis, with a median survival time of only 3-6 months, which is accompanied by clinical manifestations such as refractory ascites and abdominal wall nodules [4]. Therefore, searching for key molecular mechanisms and targets driving the intraperitoneal implantation of GC cells is of great significance for the personalized treatment of patients with GCPM.

The “seed-soil” hypothesis is considered a recognized theoretical mechanism for GCPM progression [5]. Primary GC cells invade the serosa and colonize the mammary plaques and lymphatic pores of the peritoneal mesothelium. A tumor microenvironment favorable for cancer cell metastasis is established by changing the morphology and function of peritoneal mesothelial cells, enhancing the adhesion and invasion ability of free cancer cells, and thus promoting GCPM progression. The free tumor cells in the abdominal cavity play a crucial role as “seeds” in the peritoneal metastasis of GC. Huang et al. employed single-cell sequencing to reveal the dynamic changes in free tumor and immune cells in the ascites of patients with GC during peritoneal metastasis, providing effective strategies for treating peritoneal metastasis [6]. Previous studies have confirmed that tumor cells can stimulate peritoneal mesothelial cells to undergo MMT, resulting in morphological changes in these cells. During this process, E-cadherin expression decreases whereas N-cadherin expression increases, leading to the transformation of these cells into CAFs [7]. Meanwhile, activated TGF-β1, as a soluble factor, diffuses into tumor tissues through autocrine or paracrine pathways, thereby promoting tumor cell invasion, metastasis, and immune escape [8, 9]. TGF-β1 is known as an MMT inducer, but its mechanism of inducing MMT in mesothelial cells remains unclear. The preliminary finding of the research group was that the TGF-β1 protein secreted by GC cells increased the expression of collagen III in the extracellular matrix around CAFs, thus providing a rich “soil” for the progression of peritoneal metastasis [10]. However, the mechanism of MMT-induced GCPM in mesothelial cells remains unclear.

The microfiber-related protein family is a key component of the extracellular matrix, which plays a role in various biological processes related to cell motility. Microfiber-associated protein 2 (MFAP2) is the main antigen of elastin-associated microfibers and may be involved in hereditary connective tissue diseases. MFAP2 can participate in the proliferation, migration, invasion, and regulation of tumor-related miRNA expression in tumor cells, playing numerous roles in tumor progression [11, 12]. Available data showed that MFAP2 in GC cells activated FAK/ERK by regulating the expression of integrin β1 and integrin α5, thereby promoting GC cell movement and adhesion [13]. MFAP2 enhanced the binding between FAK and integrin β1 and activated the FAK–Akt signaling pathway, thereby promoting lung metastasis in esophageal squamous cell carcinoma mouse models [14]. However, the effect of MFAP2 synthesis and secretion by CAFs on the GCPM has not yet been reported.

This study found that GC cells infiltrated and penetrated the serosa membrane and were located freely in the abdominal cavity. GC cells secreted TGF-β1 to induce MMT in peritoneal mesothelial cells, converting these cells into CAFs. Moreover, TGF-β1 secretion stimulated the entry of Smad4 into the nucleus of peritoneal mesothelial cells, promoting the synthesis and secretion of MFAP2. This altered the peritoneal microenvironment and promoted the colonization of GC cells on the peritoneum. The MFAP2 secreted by CAFs bound to the integrin αVβ3 receptor on the surface of GC cells, activating the expression of PTK7 through the Src-STAT3 axis transcription to accumulate intracellular β-catenin. This enhanced the invasion, migration, and adhesion ability of GC cells, thereby promoting GCPM progression.

## Results

### Positive expression of MFAP2 derived from CAFs in GCPM detected by single-cell sequencing analysis

**Fig. 1:**
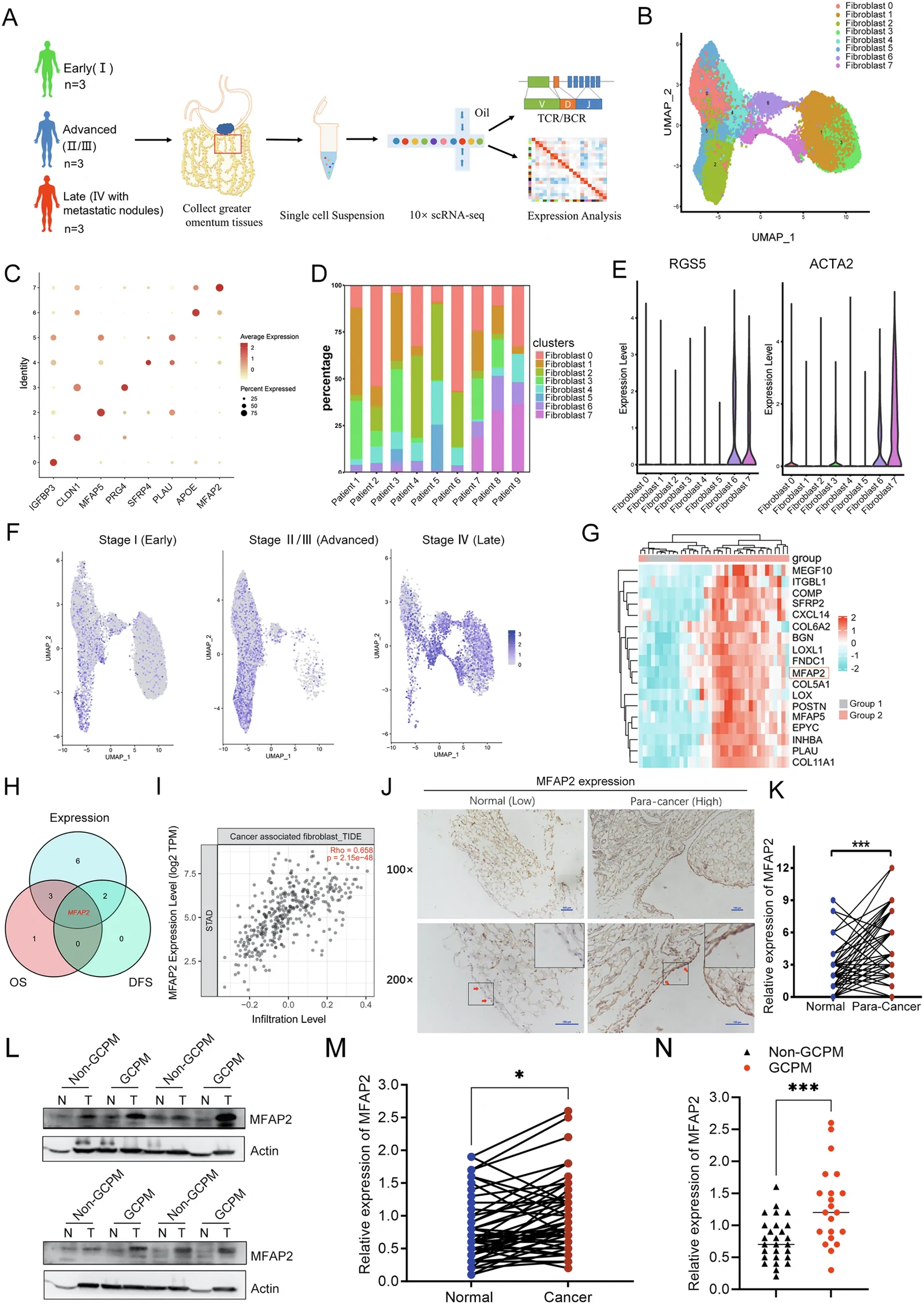
Positive expression of MFAP2 derived from CAFs in GCPM by single-cell sequencing analysis. **A** Distribution map of UMAP in fibroblasts obtained from single-cell sequencing of peritoneal tissue in nine patients with GC. **B** Fibroblasts were enriched according to expression levels, resulting in eight cell clusters (zero to seven clusters). **C** Key genes were identified for eight cell clusters. **D** Expression ratio of eight cell clusters in peritoneal tissue. **E** Expression of the CAF markers RGS5 and ACTA2 in eight cell clusters. **F** Expression of MFAP2 in different stages. **G** Heatmap showing differential expression of exocrine proteins in the human CAF tissue database GES40595. Group1 represents human normal fibrous tissue, whereas Group2 represents human tumor-related fibrous tissue. **H** Venn plot shows that the expression, overall survival (OS), and disease-free survival of the exocrine proteins mentioned earlier had statistical significance in TCGA gastric cancer data analysis. **I** Correlation between the expression of MFAP2 in the TIMER database and the proportion of CAFs. **J** Representative image of immunohistochemistry (IHC) staining. The area indicated by the arrow is enlarged in the upper right corner of the image, with an original magnification of 100×. **K** Expression of MFAP2 in normal mesothelial tissue and paracancerous peritoneal mesothelial tissue. The relative staining of all samples is shown in the scatter plot (t-test, ***p < 0.001). **L** MFAP2 expression in 48 clinical gastric cancer tissue pairs. Lysates of tumor tissues (T) and matched adjacent noncancerous tissues (**N**) were analyzed using Western blotting. Twelve representative pairs are shown. **M** The indicated protein levels in (**L**) were statistically analyzed (*p < 0.05). **N** Comparison of MFAP2 expression in GCPM (*n* = 21) and non-GCPM (*n* = 27) tissues.***p < 0.001.

We selected peritoneal tissues from nine patients with GC (three with stage I, three with stage II/III, and three with stage IV peritoneal metastasis) for single-cell sequencing analysis to investigate how alterations in mesothelial tissue in the abdominal microenvironment affect GCPM (Fig. 1A). Eight fibroblast clusters (zero to seven clusters) were enriched based on the gene expression levels and separately marked using IGFBP3, CLDN1, MFAP5, PRG4, SFRP4, PLAU, APOE, and MFAP2, respectively (Fig. 1B, C). Fibroblasts 6 and 7 were upregulated in stage IV GC tissues with peritoneal metastasis and identified as CAFs using CAF markers RGS5 and ACTA2 (Fig.  1D, E). The single-cell sequencing results showed that the number of MFAP2-positive cells in Fibroblasts 6 and 7 gradually increased with tumor progression (Fig. 1F). Then, differentially expressed secretory protein genes were selected using the human tumor-related fibroblast tissue database GES40595. Based on the findings, combined with the gene expression and survival differences in the Cancer Genome Atlas (TCGA) GC database, *MFAP2* was selected as the research target (Fig. 1G, H). In addition, TCGA database analysis in TIMER 2.0 (http://timer.cistrome.org/) revealed a positive correlation between MFAP2 expression and the proportion of CAFs, suggesting a crucial role of high MFAP2 expression in CAFs in GCPM (Fig. 1I).

We performed immunohistochemical experiments in clinical samples, revealing that the MFAP2 expression in peritoneal mesothelial tissue adjacent to cancer tissues was higher than that in normal mesothelial tissue (Fig. 1J, K). At the same time, MFAP2 expression was detected in 48 pairs of freshly paired GC and adjacent tissues. MFAP2 was highly expressed in GC tissue compared with adjacent tissues, and the expression was higher in patients with peritoneal metastasis than in those without peritoneal metastasis. (Fig. 1L–N). Meanwhile, we also analyzed MFAP2 expression in 207 GC tissues and 76 adjacent tissues through immunohistochemical experiments. We found that MFAP2 expression was higher in cancer tissues than in adjacent tissues. (Fig. S1A, S1B). The survival analysis showed that patients with high MFAP2 expression had significantly lower survival time than those with low expression (Fig. S1C). Analysis of clinical pathological factors revealed a significant correlation between MFAP2 expression level and tumor size, Borrmann type, and TNM stage (Table S1). These results suggested the crucial role of high MFAP2 expression in GCPM.

### Secretion of MFAP2 mainly by CAFs promoted the colonization of GC cells

**Fig. 2:**
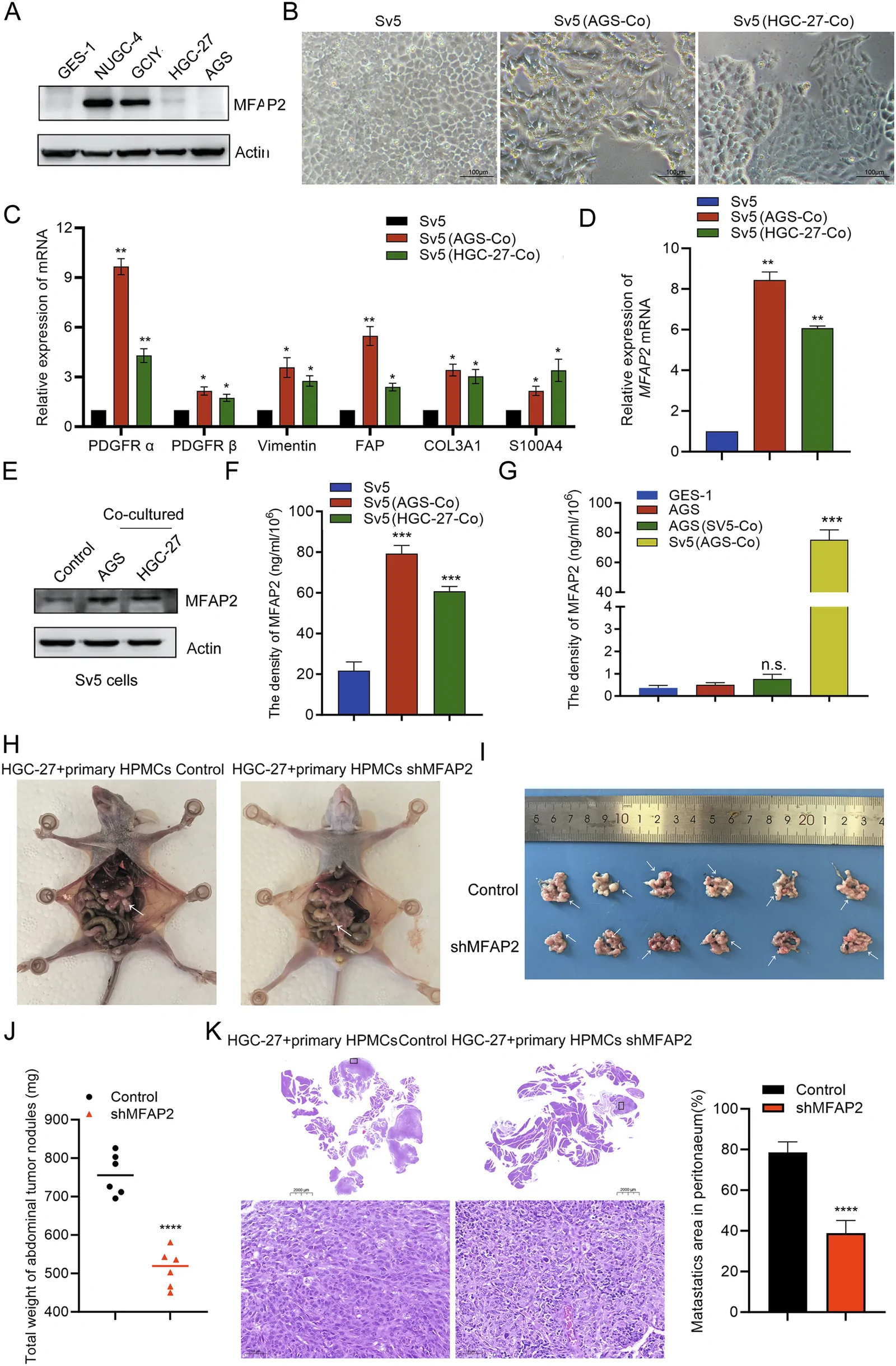
Secretion of MFAP2 mainly originated from CAFs and promoted the colonization of gastric cancer cells. **A** Expression levels of MFAP2 in different GC cell lines were determined using western blotting. **B** Morphological changes in mesothelial cells after co-cultured with GC cells indicated in the figure for 48 h. **C** qRT-PCR analysis of the CAF-associated markers in Sv5 cells co-cultured with GC cells. The levels of all mRNAs were standardized using GAPDH. The data are displayed as histograms of the average SEM of three independent experiments. The error bar represents the mean ± standard deviation; *p < 0.05, **p < 0.01, t-test. **D** qRT-PCR analysis of MFAP2 expression levels in mesothelial cells co-cultured with GC cells for 48 h. The levels of all mRNAs were standardized using GAPDH The data are displayed as histograms of the average SEM of three independent experiments. The error bar represents the mean ± standard deviation; **p < 0.01, t-test. **E** After co-culturing with the GC cells for 48 h, western blotting was performed to detect the protein level of MFAP2 in Sv5 cells. **F** After co-culturing with the GC cells for 48 h, the culture medium was collected, and ELISA was used to determine the level of MFAP2 in the culture medium. The data are displayed as histograms of the average SEM of three independent experiments. The error bar represents the mean ± standard deviation; ***p < 0.001, t-test. **G** Culture media containing GES1, AGS, AGS (co-cultured with SV5), and Sv5 (co-cultured with AGS), and the level of MFAP2 in the media using ELISA. The data are displayed as histograms of the average SEM of three independent experiments. The error bar represents the mean ± standard deviation; ***p < 0.001, t-test. **H** Control or shMFAP2 primary mesothelial cells and tumor cells were injected into the abdominal cavity (6 mice per group). The arrow in the representative image indicates the tumor. **I** Comparison of the volume of omental cancer nodules in nude mice. **J** Statistical chart of the total weight of omental cancer nodules in nude mice. ****p < 0.0001. **K** Hematoxylin and eosin staining of omental cancer nodules. On the right is the proportion of tumor cells in the metastatic nodule. ****p < 0.0001.

We first detected MFAP2 expression in normal human gastric epithelial cells and GC cell lines to investigate the reasons for the increased MFAP2 expression in the mesothelial and GC tissues of the abdominal microenvironment. The results showed low expression of MFAP2 in HGC-27 and AGS cell lines compared with NUGC-4 and GCIY cell lines (Fig. 2A). So, we co-cultured HGC-27 and AGS GC cells with mesothelial cells (Sv5) for 48 hours. We found that the morphology of Sv5 cells changed from oval to spindle-shaped (Fig. 2B) and CAF markers were upregulated (Fig. 2C), confirming the transformation of Sv5 into CAFs. The mRNA, protein, and exocrine levels of MFAP2 significantly increased in co-cultured Sv5 cells compared with the control cells (Fig. 2D–F). We co-cultured GC cells with mesothelial cells (Sv5) for 48 hours, then changed the culture medium, and cultured the cells separately for 48 hours to further determine the source of MFAP2 secretion in the culture medium. The MFAP2 level in the culture medium was detected using an enzyme-linked immunosorbent assay. The results showed that the co-cultured Sv5 cell culture medium had a higher MFAP2 level than the co-cultured tumor cells (Fig. 2G), indicating that when mesothelial cells transformed into CAFs (co-cultured Sv5 cells), the expression and exocrine level of MFAP2 in CAFs increased; the secretion of MFAP2 mainly originated from CAFs.

Next, we isolated adherent primary mesothelial cells from malignant ascites of gastric cancer patients with peritoneal metastasis (Fig. S1D, S1E). We knocked down MFAP2 in primary mesothelial cells and mixed them with HGC-27 cells for intraperitoneal injection into nude mice (Fig. 2H). On the 29th day of tumor formation, the mice were euthanized, and the tumor tissue was taken out for weighing. The results showed that compared to the control group, the tumors in the shMFAP2 group were significantly reduced, and the area of gastric cancer metastases in the tumor tissue was significantly reduced (Fig. 2I–K). We also knocked down MFAP2 in mesothelial cells and mixed it with HGC-27 cells to construct a nude mouse model of peritoneal metastasis so as to verify the effect of MFAP2 secretion from CAFs on peritoneal metastasis in GC. The mice were euthanized 4 weeks later, revealing that knocking down MFAP2 significantly reduced the number of peritoneal cancer nodules and the total weight (Fig. S1F, G). Hematoxylin and eosin staining showed that the MFAP2 knockdown group had a lower proportion of tumor cell area in the peritoneal tumor tissue of mice compared with the control group (Fig. S1H). The aforementioned results indicated that MFAP2 secretion mainly originated from CAFs in the abdominal microenvironment, which promoted the colonization of GC cells.

### TGF-β1 secretion from GC cells stimulated the entry of Smad4 into the nucleus, promoting MFAP2 transcription in peritoneal mesothelial cells

**Fig. 3:**
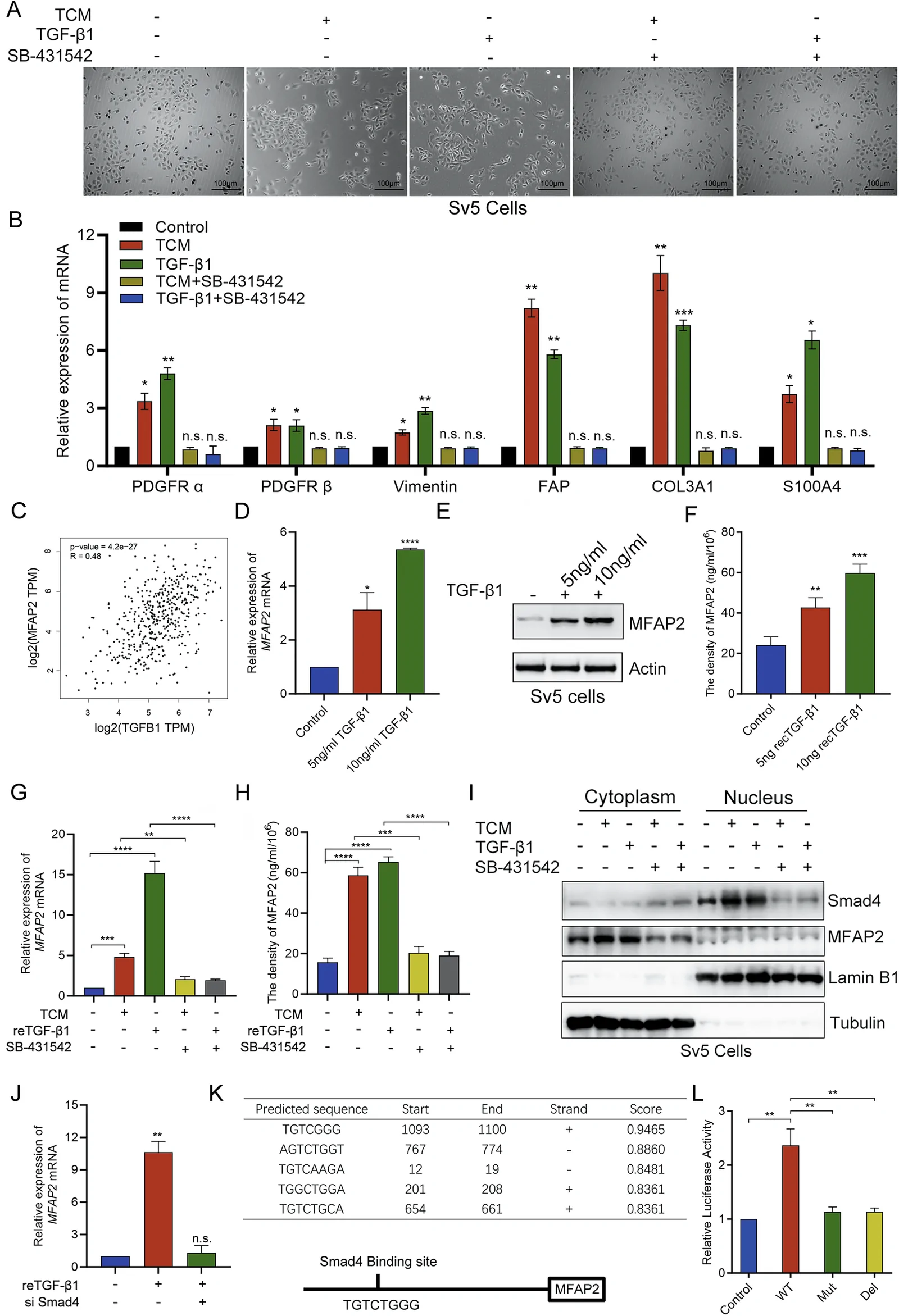
GC cells secrete TGF-β1 to stimulate intracellular Smad4 entering nuclear, promoting transcription of MFAP2 in peritoneal mesothelial cells. **A** Morphological changes in Sv5 cells treated with TCM, TGF-β1, or SB-431542 in the figure for 48 h. **B** qRT-PCR analysis of the CAF-associated markers in Sv5 cells treated with TCM, TGF-β1 or SB-431542. The levels of all mRNAs were standardized using GAPDH. The data are displayed as histograms of the average SEM of three independent experiments. The error bar represents the mean ± standard deviation; *p < 0.05, **p < 0.01, ***p < 0.001, t-test. **C** Analysis of the correlation between TGF-β1 and MFAP2 expression in TCGA gastric cancer databases. **D** qRT-PCR analysis of MFAP2 expression levels in Sv5 cells with the addition of recombinant protein TGF-β1. The levels of all mRNAs were standardized using GAPDH. The data are displayed as histograms of the average SEM of three independent experiments. The error bar represents the mean ± standard deviation; *p < 0.05, **** *p* < 0.0001, t-test. **E** Expression level of MFAP2 in Sv5 cells determined using western blotting with the addition of recombinant protein TGF-β1. **F** Recombinant protein TGF-β1 was added, and ELISA was used to determine the level of MFAP2 in the culture medium. The data are displayed as histograms of the average SEM of three independent experiments. The error bar represents the mean ± standard deviation; **p < 0.01, ***p < 0.001, t-test. **G** qRT-PCR detection of MFAP2 mRNA levels in Sv5 cells treated with TCM, TGF-β1 or SB-431542. The level of MFAP2 mRNA was standardized using GAPDH. The data are displayed as histograms of the average SEM of three independent experiments. The error bar represents the mean ± standard deviation; **p < 0.01, ***p < 0.001, **** *p* < 0.0001, t-test. **H** Sv5 cells treated with TCM, TGF-β1 or SB-431542. ELISA was used to determine the level of MFAP2 in the culture medium. The data are displayed as histograms of the average SEM of three independent experiments. The error bar represents the mean ± standard deviation; ***p < 0.001, ****p < 0.0001, t-test. **I** Separation of cytoplasmic protein and nuclear protein with nucleoplasm separation Kit. Western blotting with the indicated antibodies in Sv5 cells treated with TCM, TGF-β1, or SB-431542. **J** qRT-PCR analysis of MFAP2 expression levels in Sv5 cells by adding recombinant protein TGF-β1 or knocking down Smad4 as shown in the figure. The level of MFAP2 mRNA was standardized using GAPDH. The data are displayed as histograms of the average SEM of three independent experiments. The error bar represents the mean ± standard deviation; **p < 0.01, t-test. **K** Jasper predicted the binding sequence of Smad4 and MFAP2. **L** Transfection of control, MFAP2 WT, MFAP2 Mut, MFAP2 Del, and Smad4 into mesothelial cells, and detection of MFAP2 expression through luciferase reporter gene assay (t-test, **p < 0.01).

When the primary lesion of gastric cancer has not undergone significant metastasis, the peritoneal microenvironment has undergone significant changes. To identify the key factors secreted by GC cells that promote MMT in mesothelial cells, we performed 4D-DIA quantitative proteomics on peritoneal lavage fluid and supernatant of serum-free cultured cancer cells from early- and late-stage cancer patients. We also performed cross-analysis on the top 150 upregulated secreted proteins and identified 15 upregulated proteins (Fig. S2A). Among these, TGF-β1 is a widely recognized MMT inducer to promote the MMT phenotype in mesothelial cells [15]. We used the TCGA GC database to analyze the association between TGF-β1 and malignant progression, metastasis, and poor prognosis of GC (Fig. S2B–S2D). We co-cultured GC cells with mesothelial cells or added TGF-β1 alone. We discovered changes in the morphology of mesothelial cells and upregulated expression of CAF markers. However, when the TGF-β1 inhibitor (SB-431542) was added, no significant changes in the morphology of mesothelial cells and the expression of CAF markers were observed, indicating that GC cells induced the transformation of mesothelial cells into CAFs by secreting TGF-β1 (Fig. 3A, B). GSEA enrichment analysis showed that MFAP2 was highly enriched in the TGF-β1 pathway (Fig. S2E). The analysis of the TCGA and Gene Expression Omnibus GC databases showed a positive correlation between TGF-β1 and MFAP2 expression (Fig. 3C and Fig. S2F). We gradually added purified TGF-β1 protein to mesothelial cells to induce their transformation into CAFs so as to verify whether the increase in synthesis and secretion of MFAP2 in CAFs was related to TGF-β1 induction. Then, we detected the mRNA and protein expression of MFAP2 in cells, as well as the secretion level of MFAP2 in the culture medium. The results showed an increase in mRNA, protein, and exocrine levels of MFAP2 in CAFs (Fig. 3D–F). After adding SB-431542, the increase in mRNA and exocrine levels of MFAP2 induced by TGF-β1 in mesothelial cells was reversed (Fig. 3G, H), suggesting that TGF-β1 might be a key factor leading to the increase in MFAP2 expression and secretion level in CAFs.

TGF-β1 can stimulate Smad4 to enter the nucleus and regulate target gene transcription [16, 17], but the effect of Smad4 on MFAP2 has not been reported yet. We co-cultured mesothelial cells with GC cells or induced them with TGF-β1 protein alone. We found a significant increase in the entry of Smad4 into the nucleus. On adding SB-431542, the entry of Smad4 into the nucleus was inhibited (Fig. 3I). We silenced Smad4 in mesothelial cells and induced the transformation of these cells into CAFs by adding TGF-β1 to determine that Smad4 was an upstream transcription factor of MFAP2. We found that silencing Smad4 significantly reduced the mRNA level of MFAP2 in CAFs compared with the control group (Fig. 3J). Next, potential binding sites between Smad4 and the MFAP2 promoter were predicted using the JASPAR database (Fig. 3K), and the highest-scoring binding site was selected to construct its WT. Mut and Del plasmids were used to confirm through luciferase experiments that Smad4 was a potential upstream transcription factor of MFAP2 (Fig. 3L).

### MFAP2 increased the adhesion and invasion ability of GC cells

**Fig. 4:**
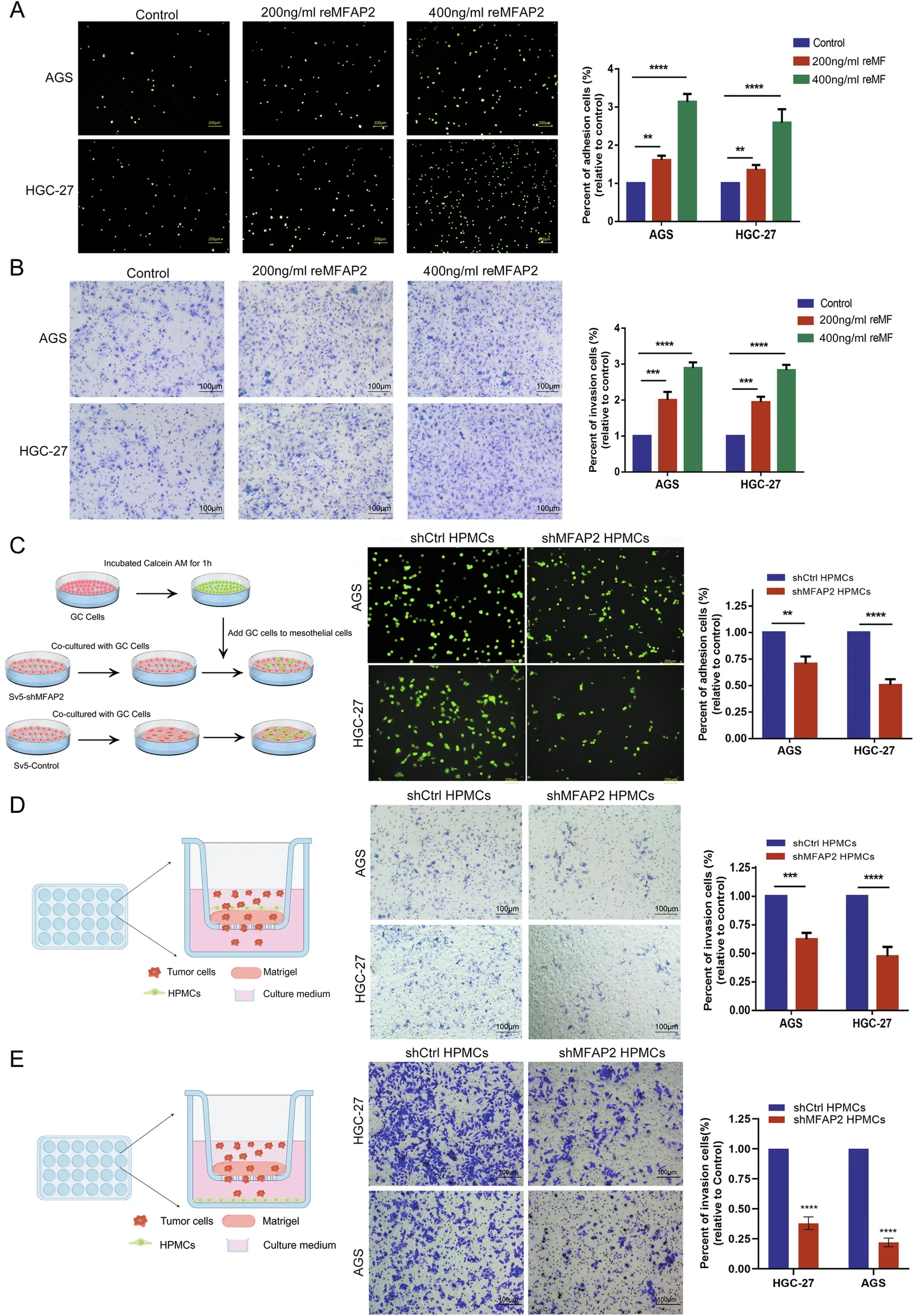
MFAP2 increased the adhesion and invasion ability of GC cells. **A** Adhesion analysis of AGS (upper) or HGC27 (lower) cells with added recombinant protein MFAP2. On the right is the statistical histogram, and the results of three independent experiments are representative, with **p < 0.01 and ****p < 0.0001. **B** Transwell analysis of AGS (upper) or HGC27 (lower) cells with added recombinant protein MFAP2. On the right is the statistical histogram. The results of three independent experiments are representative, with ***p < 0.001 and ****p < 0.0001. **C** Co-cultured of control or shMFAP2 mesothelial cells and tumor cells for 48 h; adhesion analysis of AGS (upper) or HGC27 (lower) cells, with statistical histograms on the right. The results of three independent experiments are representative, with **p < 0.01 and ****p < 0.0001. **D** Co-cultured of control or shMFAP2 mesothelial cells and tumor cells, Transwell analysis of AGS (upper) or HGC27 (lower) cells, and statistical histogram on the right. The results of three independent experiments are representative, with ***p < 0.001 and ****p < 0.0001. **E** Co-cultured of control or shMFAP2 mesothelial cells and tumor cells, Transwell indirect system analysis of AGS (lower) or HGC27 (upper) cells, and statistical histogram on the right. The results of three independent experiments are representative, with ****p < 0.0001.

To further investigate the effect of MFAP2 secreted by CAFs on the function of gastric cancer cells. we gradually added purified protein MFAP2 (reMFAP2) to AGS and HGC-27 cells to verify the effect of MFAP2 on the adhesion and invasion ability of GC cells. We found that the adhesion and invasion ability of GC cells gradually increased with the increase in MFAP2 concentration compared with that in the control group (Fig. 4A, B). Moreover, knocking down MFAP2 in mesothelial cells and co-culturing these cells with tumor cells revealed that MFAP2 knockdown significantly reduced the adhesion ability of GC cells compared with that in the control group (Fig. 4C). We used transwell experiments to simulate the invasion of GC cells into the peritoneal mesothelial layer in vitro, and confirmed that knocking down MFAP2 in mesothelial cells inhibits the invasion of GC cells (Fig. 4D). To verify that MFAP2 acts on gastric cancer cells through exocrine means, We knocked down MFAP2 from mesothelial cells and planted it on a well plate below the transwell chamber to observe its effect on the invasion ability of GC cells. We found that compared with the control group, knocking down MFAP2 significantly reduced the invasion ability of GC cells (Fig. 4E).

### Integrin αVβ3 was a key receptor for MFAP2 in GC cells

**Fig. 5:**
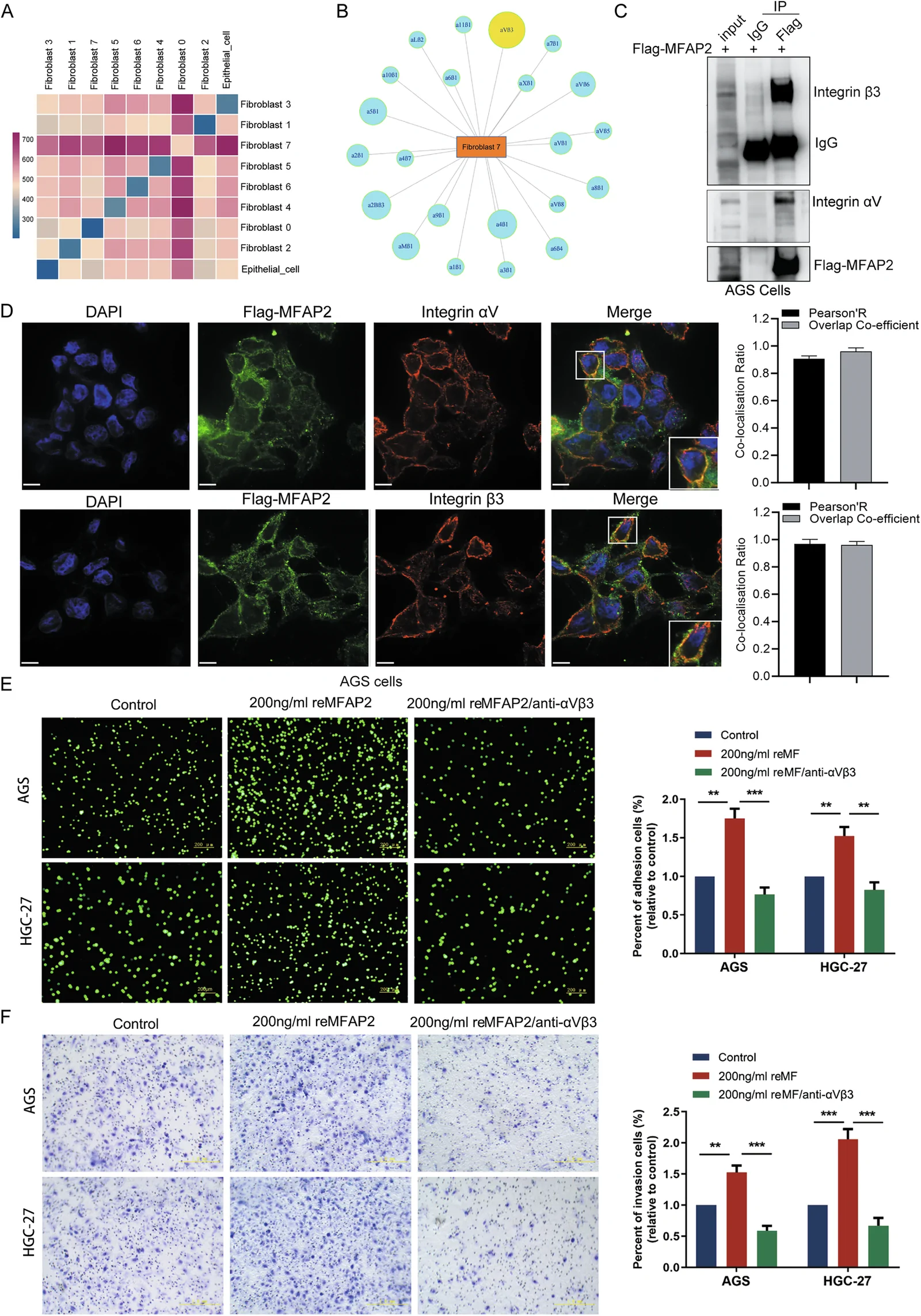
Integrin αVβ3 was a key receptor for MFAP2 in GC cells. **A** Crosstalk plot of epithelial cells and eight clusters of fibroblasts. **B** Ligand receptor-related network of interactions between fibroblast Cluster 7 and epithelial cells. The nodes in the graph represent different receptors, whereas the node size represents the strength of their interaction relationship. **C** AGS cells co-cultured with Sv5 cells transfected with Flag-MFAP2 and cells for 48 hours. IP and IB analyses of the interaction between MFAP2 and integrin β3 or integrin αV. Western blotting with the indicated antibodies. **D** AGS cells co-cultured with Sv5 cells transfected with Flag-MFAP2 for 48 hours. Immunofluorescence staining of AGS cells for MFAP2 and integrin β3 or integrin αV. Original magnification was 600×. **E** Adhesion analysis of AGS (upper) or HGC27 (lower) cells with added recombinant protein MFAP2 and anti-Integrin αVβ3. On the right is the statistical histogram, and the results of three independent experiments are representative, with **p < 0.01 and ***p < 0.001. **F** Transwell analysis of AGS (upper) or HGC27 (lower) cells with added recombinant protein MFAP2 and anti-Integrin αVβ3. On the right is the statistical histogram, and the results of three independent experiments are representative, with **p < 0.01 and ***p < 0.001.

The integrin family is considered to be the receptor for most cytokines and matrix proteins. Module interaction analysis revealed that Fibroblast 7 had the strongest association with epithelial receptor ligands. Also, we identified integrin αVβ3 as the most crucial receptor associated with Fibroblast 7 (Fig. 5A, B and Fig. S3). MFAP2 was first overexpressed in Sv5 cells and co-cultured with GC cells for 48 hours to investigate whether MFAP2 bound to the integrin αVβ3 receptor in GC cells. Immunoprecipitation was used to confirm the interaction between MFAP2 and integrin αV/integrin β3 in GC cells (Fig. 5C). Confocal laser microscopy confirmed the co-localization of MFAP2 with integrin αV and integrin β3 on the cell membrane (Fig. 5D), indicating integrin αVβ3 as the cellular receptor of MFAP2. Purified MFAP2 protein and inhibitors of integrin αVβ3 were subsequently added to GC cells to investigate whether MFAP2 promotes GC progression by acting on GC cells through integrin αVβ3. Cell adhesion and invasion assays showed that adding integrin αVβ3 inhibitors could reverse the promotive effects of purified MFAP2 on GC cell adhesion and invasion (Fig. 5E and F).

### MFAP2 secreted by CAFs promoted the adhesion and invasion ability of GC cells through the Src/STAT3/PTK7/β-catenin signaling pathway

**Fig. 6:**
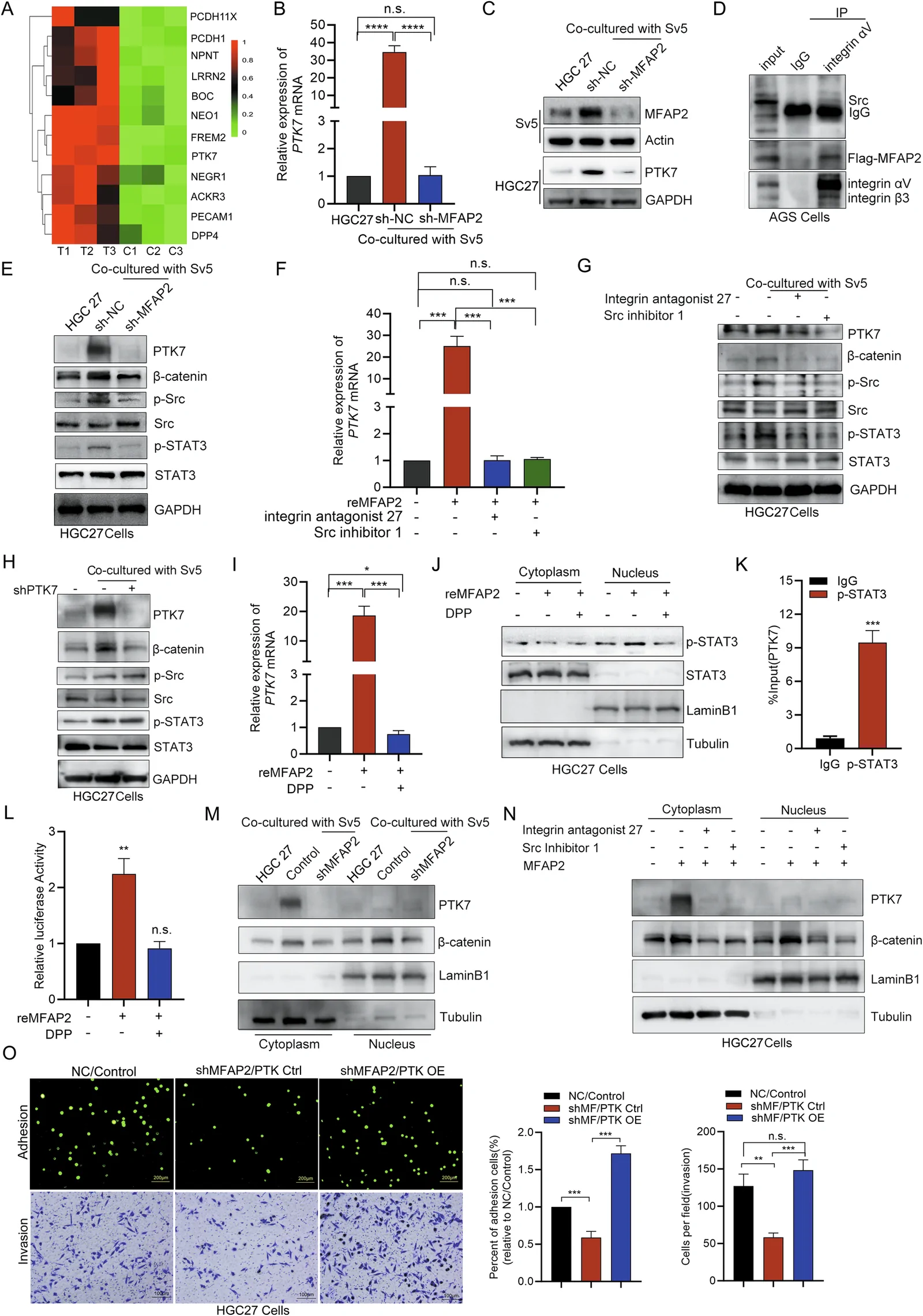
MFAP2 secreted by CAFs promoted GC cells adhesion and invasion through the Src/STAT3/PTK7/β-catenin signaling pathway. **A** Heatmap of enriched genes in cell adhesion pathway. **B** Relative levels of PTK7 were detected in HGC27 cells alone or after co-culture with Sv5, in which MFAP2 was knocked down. The level of PTK7 mRNA was standardized using GAPDH. The data are displayed as histograms of the average SEM of three independent experiments. The error bar represents the mean ± standard deviation; ****p < 0.0001, t-test. **C** HGC27 cells co-cultured with Sv5 cells transfected with control or shMFAP2 for 48 h. Western blotting with the indicated antibodies. **D** AGS cells co-cultured with Sv5 cells transfected with Flag-MFAP2 for 48 hours. IP and IB analyses of the interaction between MFAP2, Src, integrin β3 and integrin αV. Western blotting with the indicated antibodies. **E** Immunoblotting to assess activation of the Src-STAT3-PTK7 signaling pathway in HGC27 cells alone or co-cultured with Sv5, in which MFAP2 was knocked down. **F** qRT-PCR assays of PTK7 expression in HGC27 cells treated with reMFAP2, integrin antagonist 27 or Src inhibitor 1 compared to controls. The level of PTK7 mRNA was standardized using GAPDH. The data are displayed as histograms of the average SEM of three independent experiments. The error bar represents the mean ± standard deviation; ***p < 0.001, t-test. **G** Immunoblotting to assess activation of the Src-STAT3-PTK7 signaling pathway in HGC27 cells alone or co-cultured with Sv5, and treated with integrin antagonist 27 or Src inhibitor 1. **H** Transfection of control or shPTK7 into GC cells. Immunoblotting to assess activation of the Src-STAT3-PTK7 signaling pathway in HGC27 cells alone or co-cultured with Sv5. **I** qRT-PCR detection of PTK7 mRNA levels in HGC27 cells treated with reMFAP2 following STAT3 inhibition with 5,15-DPP. The level of PTK7 mRNA was standardized using GAPDH. The data are displayed as histograms of the average SEM of three independent experiments. The error bar represents the mean ± standard deviation; ***p < 0.001, t-test. **J** Separation of cytoplasmic protein and nuclear protein with nucleoplasm separation Kit. Western blotting with the indicated antibodies in HGC27 cells treated with reMFAP2 following STAT3 inhibition with 5,15-DPP. **K** ChIP-qPCR assay to confirm that p-STAT3 is the transcription factor for PTK7, ***p < 0.001. **L** HGC27 cells treated with reMFAP2 following STAT3 inhibition with 5,15-DPP, and detection of PTK7 expression through luciferase reporter gene assay (t-test, **p < 0.01). **M** Separation of cytoplasmic protein and nuclear protein with Nucleoplasm Separation Kit. Western blotting with the indicated antibodies in HGC27 cells alone or co-cultured with Sv5, in which MFAP2 was knocked down. **N** Separation of cytoplasmic protein and nuclear protein with nucleoplasm separation Kit. Western blotting with the indicated antibodies in HGC27 cells treated with reMFAP2, integrin antagonist 27, or Src inhibitor 1. **O** Transfection of control or shMFAP2 into mesothelial cells and control or PTK into GC cells, co-cultured for 48 hours, followed by adhesion/invasion analysis. Statistical histogram of cell adhesion/invasion analysis in (M) shows that the results of three independent experiments are representative (**p < 0.01, ***p < 0.001).

We stimulated GC cells with purified MFAP2 protein and performed transcriptome sequencing to screen for genes regulated by MFAP2 (Fig. S4A). Gene Ontology (GO) enrichment analysis was performed on the differentially expressed genes between the two groups. The results showed a crucial role of MFAP2 in the composition of the extracellular matrix and cell adhesion (Fig. S4B). We extracted genes from the cell adhesion module and performed GSEA analysis on these genes, further confirming the critical role of MFAP2 in GC cell adhesion (Fig. S4C). We crossed genes related to cell adhesion, invasion, and metastasis pathways to obtain the downstream target PTK7 of MFAP2 (Fig. 6A). The correlation analysis of the TCGA cancer database showed a positive correlation between the expression levels of MFAP2 and PTK7 (Fig. S4D). We knocked down MFAP2 in mesothelial cells and co-cultured them with GC cells. The mRNA level and protein expression of PTK7 in GC cells were significantly reduced (Fig. 6B, C). PTK7 exerts a pro-tumor effect by accumulating β-catenin [18, 19]. Therefore, we knocked down MFAP2 in mesothelial cells and co-cultured these cells with GC cells. We found a significant decrease in the expression of β-catenin in tumor cells. However, PTK7 overexpression in tumor cells reversed the regulatory effect of MFAP2 knockdown on β-catenin. (Fig. S4E).

The activation of integrin αVβ3 is related to the activation of Src kinase signaling transduction, and STAT3 is a key Src kinase effector [20–22]. We hypothesize that MFAP2 regulates PTK7 expression through the integrin αVβ3-Src-STAT3 pathway. To explore this hypothesis, MFAP2 was overexpressed in Sv5 cells and co-cultured with GC cells for 48 hours. Co-immunoprecipitation confirmed that the membrane receptor integrin αVβ3 of MFAP2 interacts with intracellular Src to activate Src kinase signaling transduction (Fig. 6D). We assessed protein expression across the suspected signaling cascade in GC cells and mesothelial-GC cell co-cultures. The shMFAP2 group showed decreased protein levels of p-Src, p-STAT3, PTK7, and β-catenin (Fig. 6E and S4F). Stimulation of GC cells with purified MFAP2 protein revealed an increase in mRNA and protein levels of PTK7. The addition of integrin αVβ3 inhibitor (integrin antagonist 27) and Src inhibitor (Src inhibitor 1) reversed MFAP2-induced activation of Src signaling, PTK7 expression, and downstream changes in β-catenin expression (Fig. 6F, 6G and S4G). Further, knocking down PTK7 in GC cells and co-culturing these cells with mesothelial cells to induce activation of Src signaling did not lead to intracellular accumulation of β-catenin (Figs. 6H and S4H). To verify the transcriptional regulation of PTK7 by STAT3, purified MFAP2 protein was used to stimulate GC cells to activate the Src-STAT3 signaling pathway and upregulate PTK7 expression. STAT3 inhibition with 5,15-diphenylporphyrin (DPP) reversed the upregulation of PTK7 induced by MFAP2 (Fig. 6I). Nuclear-cytoplasmic separation experiments confirmed that MFAP2 stimulated GC cells to activate the Src-STAT3 signaling pathway, the entry of p-STAT3 into the nucleus significantly increased (Fig. 6J). The potential binding sites between p-STAT3 and the PTK7 promoter were predicted using the JASPAR database, and the highest scoring binding site was selected for ChIP-qPCR and luciferase assay to confirm that p-STAT3 is the potential upstream transcription factor for PTK7 (Figs. 6K, L and S4I). Nuclear-cytoplasmic separation experiments confirmed that MFAP2 upregulated PTK7 expression through the integrin αVβ3-Src-STAT3 pathway, leading to downstream β-catenin accumulation and entry into the nucleus. The addition of integrin antagonist 27 and Src inhibitor 1 reversed the effects of MFAP2 (Fig. 6M, N). Cell adhesion and invasion assays further confirmed that knocking down MFAP2 in mesothelial cells suppressed the adhesion and invasion ability of co-cultured GC cells. PTK7 overexpression in GC cells reversed the inhibitory effect of shMFAP2 on GC cell adhesion (Figs. 6O and Fig. S4J).

### MFAP2 was positively correlated with PTK7 expression in GC tissue samples and associated with poor prognosis

**Fig. 7:**
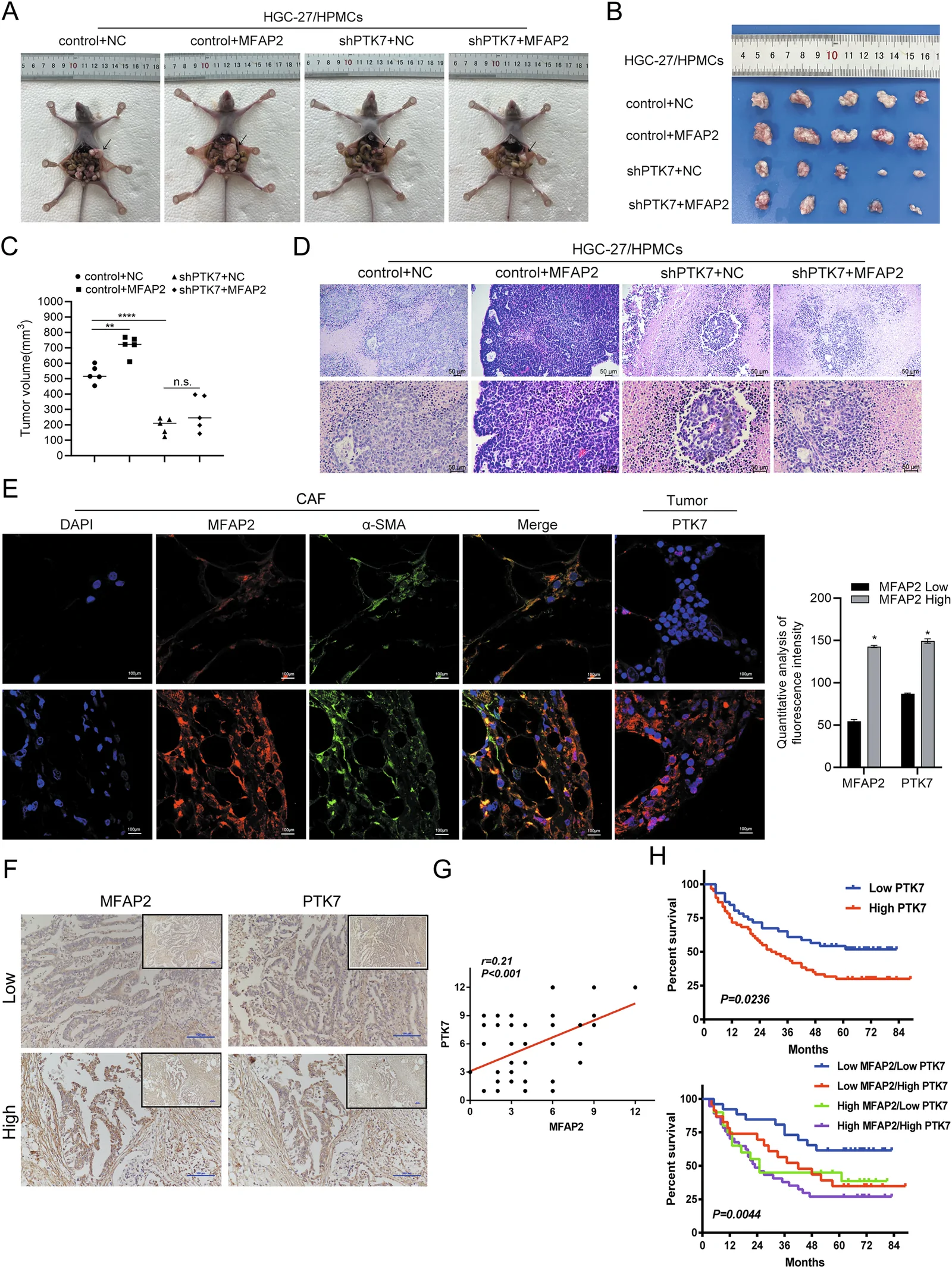
MFAP2 was positively correlated with PTK7 expression in tissue samples of GC and associated with poor prognosis. **A** Nude mouse intraperitoneal injection model in vivo. The samples were divided into four groups: HGC27 Control/Sv5 NC, HGC27 Control/Sv5 MFAP2, HGC27 shPTK7/Sv5 NC, and HGC27 shPTK7/Sv5 MFAP2 (5 mice per group). **B** Comparison of the volume of omental cancer nodules in nude mice. **C** Statistical chart of the volume of omental cancer nodules in nude mice (**p < 0.01 and ****p < 0.0001). **D** Hematoxylin and eosin staining of omental cancer nodules. **E** Immunofluorescence detection of MFAP2 and PTK7 expression in peritoneal metastatic nodules of type IV GC. On the right is the statistical histogram (*p < 0.05). **F** Representative images of IHC staining. The boxed areas in the images are magnified in the bottom images. Original magnification was 100×. **G** IHC staining was scored, and a Pearson correlation test was performed. Note that the scores for some samples overlapped. **H** Kaplan-Meier plot of OS of patients stratified by PTK7 expression levels (left). The patients were divided into different groups based on the indicated MFAP2 and PTK7 expression scores in the tumors. Kaplan–Meier plot of OS of patients stratified by MFAP2 and PTK7 expression.

A tumor formation experiment was conducted by intraperitoneal injection in nude mice to further confirm the regulatory effect of exogenous MFAP2 on PTK7 expression in GC cells. HGC-27 Control/shPTK7 were mixed with Sv5 NC/MFAP2 and injected into the abdominal cavity of nude mice (Fig. 7A). The mice were euthanized after 4 weeks. The results showed that MFAP2 overexpression in Sv5 promoted the colonization and growth of GC cells on the greater omentum of nude mice, whereas knocking down PTK7 expression in HGC-27 reversed the effect of MFAP2 secreted by Sv5 on GC cells (Fig. 7B–D). We explored the correlation and clinical significance of MFAP2 and PTK7 in patients with GC, and detected MFAP2 and PTK7 expression in omental cancer nodules of 11 cases of stage IV GC using immunofluorescence. The results showed that the fluorescence intensity of PTK7 in tumor nodules increased with the increase in the expression level of MFAP2 in CAF cells (Fig. 7E). Meanwhile, we conducted immunohistochemical experiments and found a positive correlation between MFAP2 and PTK7 expression (Fig. 7F, G). Based on PTK7 expression, we conducted Kaplan-Meier survival analysis on patients and found that those with high PTK7 expression had lower survival than those with low expression. We conducted a survival analysis on further grading of patients and found that patients with high expression of both MFAP2 and PTK7 had the worst prognosis (Fig. 7H). These results indicated a positive correlation of MFAP2 with PTK7 expression and its association with poor prognosis.

## Discussion

The microenvironment of GCPM comprises a network of various cells including tumor cells, mesothelial cells, immune cells, and so forth. These complex interactions and regulatory relationships contribute to the diversity of treatment strategies for GCPM and also increase the difficulty in designing and implementing treatment plans. Mesothelial tissue serves as the first barrier against tumor cell dissemination. MMT of mesothelial cells into CAFs promotes the adhesion and fixation of GC cells. However, the mechanism by which CAFs promote GCPM remains unclear. Therefore, we performed single-cell sequencing of peritoneal tissue from nine cases with different stages of GC, and found that Fibroblasts 6 and 7 had an increased proportion of cells in stage IV GC. Also, MFAP2 was highly expressed in Fibroblasts 6 and 7, suggesting a crucial role of MFAP2 in GCPM.

Many recent studies have explored the mechanism of peritoneal metastasis in patients with GC using peritoneal lavage fluid or ascites [6, 10, 23]. Huang et al. characterized the single-cell transcriptome of 191,987 ascitic cancer/immune cells from 35 patients with GCPM. An increase in the proportion of monocyte-like dendritic cells was observed, which exhibited decreased antigen presentation ability and were associated with poor prognosis. Wang et al. conducted single-cell transcriptome analysis on peritoneal cancer in 15 patients with gastric adenocarcinoma, constructed a map of 45,048 PC cells, analyzed the transcriptome status of the tumor cell population, delved into the intratumoral heterogeneity of malignant PC cells, and observed a significant correlation with patient survival rate. Given the complexity of the peritoneal ecosystem and the unclear mechanisms underlying GCPM, collecting ascitic fluid cells alone may yield fewer informative cells and cannot fully simulate the microenvironmental changes in GCPM. Therefore, the collection of peritoneal tissue from patients with different stages of GC, including metastatic nodules, can better demonstrate GCPM progression.

The TGF-β1 protein secreted by GC cells can cause morphological changes in mesothelial cells [24]. However, the mechanism by which TGF-β1 acts on mesothelial cells to promote the adhesion and invasion of GC cells remains unclear. We found that co-culturing GC cells with mesothelial cells (Sv5) induced the transformation of mesothelial cells into CAFs, and TGF-β1 was a key factor leading to an increase in the expression and secretion of MFAP2 in CAFs. MFAP includes five subfamily members (MFAP1-5) and is an extracellular matrix glycoprotein component involved in elastin production during microfibril assembly and the stability of the tissue environment [25–29]. MFAP2 is the first to be characterized and plays a crucial role in intercellular signal transduction. Many studies have demonstrated the impact of abnormal levels of MFAP2 on prognosis in various cancers, highlighting its vital role in tumor progression, especially in epithelial-mesenchymal transition (EMT). In melanoma metastasis, MFAP2 influences the protein level of the Wnt/β-catenin junction, activates the Wnt/β-catenin signaling pathway, and enhances EMT [30]. In addition, the changes in MFAP2 levels play a crucial role in regulating tumor cell proliferation, apoptosis, migration, angiogenesis, and ECM remodeling. However, the role of MFAP2 derived from peritoneal mesothelial cells in GC progression has not been reported. The integrin family comprises transmembrane glycoproteins composed of heterodimers formed by alpha and beta subunits, which act as receptors to mediate adhesion between cells and matrix, as well as between cells [31, 32]. We confirmed the interaction between MFAP2 and integrin αVβ3 on the cell membrane, suggesting integrin αVβ3 as the receptor of MFAP2. In vitro functional experiments and nude mouse peritoneal metastasis models have shown that MFAP2 secreted by CAFs promotes GCPM progression.

We used purified MFAP2 protein to stimulate GC cells and performed transcriptome sequencing to further investigate the molecular mechanism of MFAP2 promoting peritoneal metastasis in GC cells. Gene Ontology enrichment analysis was performed on the differentially expressed genes obtained, and the genes enriched in cell adhesion-, invasion-, and metastasis-related pathways were intersected to obtain the downstream target PTK7 of MFAP2. PTK7, a single transmembrane Wnt/CCP co-receptor, is a member of the receptor tyrosine kinase pseudokinase family and contains a tyrosine kinase-like domain that catalyzes intracellular inactivation. PTK7 participates in nonclassical Wnt signaling by interacting with Wnt ligands (Wnt5a or Wnt3a), thereby promoting cell proliferation, invasion, migration, and stemness [18, 33–37]. Further, evidence suggested a significant role of PTK7 in vascular endothelial growth factor signaling/plexin and classical Wnt signaling pathways [34, 38]. PTK7 is also highly expressed in various cancers and associated with poor prognosis and tumor metastasis [39–42]. We found that MFAP2 upregulated PTK7 expression by activating the Src-STAT3 signaling pathway through integrin αVβ3 on the GC cell membrane, thereby accumulating β-catenin and enhancing the adhesion and invasion ability of GC cells. Also, we found that MFAP2 and PTK7 expression in clinical samples of GC was positively correlated with poor prognosis. These results indicated that exogenous MFAP2 promoted the adhesion and invasion of GC cells through the integrin αVβ3-Src-STAT3-PTK7-β-catenin signaling pathway.

This study found, through single-cell sequencing of nine cases of GCPM, that high MFAP2 expression in CAFs in advanced GC promoted GCPM. As shown in the graphical abstract, TGF-β1 secreted by GC cells induced the transformation of mesothelial cells into CAFs and upregulated the expression and secretion of MFAP2 in CAFs through Smad4. Finally, we found that MFAP2/integrin αVβ3 promoted GC cell invasion, migration, and adhesion by regulating the Src-STAT3-PTK7/β-catenin signaling pathway, which might be a new therapeutic strategy for GCPM.

## Materials and methods

### Patient specimens

Nine fresh peritoneal tissues from patients with GC (three cases of stage I, three cases of stage II/III, and three cases of stage IV peritoneal metastasis) were selected from the Gastric Surgery Department of Liaoning Cancer Hospital (China, Shenyang) for single-cell sequencing analysis. In addition, we collected 11 fresh samples of stage IV GC with omental cancer nodules and 40 paired cases of normal and paracancerous peritoneal tissue embedded in paraffin. Further, we collected paraffin-embedded sections from 207 GC tissues and 76 adjacent non-cancerous tissues, along with 48 fresh pairs of cancerous and adjacent tissues. The selected GC tissue samples were obtained from the Gastrointestinal Oncology Department of the First Affiliated Hospital of China Medical University (China, Shenyang). The patients underwent GC resection without prior radiotherapy or chemotherapy. All patients signed an informed consent form, and the surgical procedures followed the regulations of Liaoning Cancer Hospital & Institute.

### Tissue Dissociation and Single-Cell Suspension Preparation

Human gastric cancer peritoneal tissues were obtained and processed as follows. A sterile, RNase-free culture dish containing calcium- and magnesium-free 1× phosphate-buffered saline (PBS) was placed on ice. The tissues were transferred into the dish, cut into 0.5 mm² pieces, and washed with 1×PBS to remove non-target materials such as blood stains and fatty layers. The tissue was dissociated into single cells using a dissociation solution (0.35% collagenase IV, 2 mg/mL papain, 120 units/mL DNase I) in a 37 °C water bath with shaking at 100 rpm for 20 min. The digestion was terminated by adding 1×PBS with 10% fetal bovine serum. The mixture was pipetted 5-10 times with a Pasteur pipette. The resulting cell suspension was filtered through a 70- and 30-µm stacked cell strainer (Corning, USA) and centrifuged for 5 min at 300 *g* and 4 °C. For cell processing, red blood cells were lysed, and the dead cells were removed by adding 50 µL of 1×PBS (0.04% bovine serum albumin) to create a cell suspension. The cell viability was assessed using trypan blue staining, aiming for > 85%. The cells were counted using a hemocytometer or an automated cell counter, targeting a concentration of 700-1200 cells/µL.

### Chromium 10x genomics library and sequencing

The single-cell suspension was loaded onto a 10× Chromium system to capture 5000 single cells by using the 10× Genomics Chromium Single-Cell 3’ Kit, V3. Then, cDNA amplification and library construction were performed following the standard protocol. The libraries were sequenced using the Illumina NovaSeq 6000 system (paired-end multiplexing run, 150 bp) at LC-Biotechnology Co.Ltd. (Hangzhou, China), with a minimum depth of 20000 reads per cell.

### Bioinformatics analysis

The sequencing data were demultiplexed and converted into FASTQ format using Illumina bcl2fastq software (version 5.0.1). Demultiplexing, barcode processing, and single-cell 3’ gene counting were performed using the Cell Ranger pipeline (version 6.1.1). The scRNA-seq data were aligned to the Ensembl GRCh38/GRCm38 reference genome. A total of 74,264 single cells from 9 patients were processed using the 10x Genomics Chromium Single Cell 3’ Solution. The output from Cell Ranger was imported into Seurat (version 3.1.1) for dimensionality reduction, clustering, and analysis of the scRNA-seq data. Clustering was performed using a weighted Shared Nearest Neighbor graph-based method. The marker genes for each cluster were identified using the Wilcoxon rank-sum test with default parameters via the FindAllMarkers function in Seurat, selecting markers expressed in more than 10% of the cells within a cluster and with an average log (fold change) greater than 0.25.

### Cell culture

Human gastric cancer cell lines NUGC-4, GCIY, AGS, HGC-27, and GES-1 (gastric epithelial cells) were purchased from the Cell Culture Collection of the Chinese Academy of Sciences (Shanghai, China). The human peritoneal mesothelial cell (HPMC) line was purchased from BeNa Culture Collection (Henan, China). All cell cultures used a medium mixed with 10% fetal bovine serum (Biological Industries, Israel). The ascites samples were passed through a 70 mm filter (Falcon, Corning, NY) and centrifuged at 500 *g* for 5 minutes. Cell pellets were resuspended in 1x ACK buffer (Thermo Fisher Scientific, Waltham, MA) and incubated at room temperature for 3 min to lyse red blood cells. After washing with PBS and counting the cells, the cells were cultured in Minimum Essential Medium Eagle. Mesothelial cells were naturally selected as they were passaged more than 2 times and stored at -80 °C until further use. Confirmation of mesothelial cells extracted from ascites through mesothelial cell markers using immunofluorescence analysis.

### Western blotting

The cells were lysed in RIPA lysis buffer containing protease and phosphatase inhibitors at 4 °C for 30 min and centrifuged for 20 min at 13,000 *g* and 4 °C. Then, the whole-cell extracts were eluted in a buffer containing 1% sodium dodecyl sulfate (SDS) at 100 °C for 5 min. Finally, the proteins were separated via SDS-polyacrylamide gel electrophoresis (SDS-PAGE). The samples were incubated with anti-MFAP2 (Abcam, America, ab231344), anti-PTK7 (17799-1-AP; Proteintech, China), anti-β-catenin (66379-1-Ig; Proteintech, China), anti-Flag (66008-4-Ig; Proteintech, China), anti-SMAD4 (10231-1-AP; Proteintech, China), anti-STAT3 (10253-2-AP; Proteintech, China), anti-Phospho-STAT3 (80199-2-RR; Proteintech, China), anti-SRC (11097-1-AP; Proteintech, China), anti-Phospho-SRC (80706-3-RR; Proteintech, China), anti-Lamin B1 (12987-1-AP; Proteintech, China), anti-Tubulin (10094-1-AP; Proteintech, China) and anti-GAPDH antibodies (60004-1-Ig; Proteintech, China). Specific primary antibodies and secondary anti-mouse or anti-rabbit antibodies conjugated to horseradish peroxidase were used to perform western blotting. Chemiluminescence technology was used to visualize the results.

### Immunohistochemistry

The procedure and scoring in this experiment were carried out as previously described [43]. The dilution ratio of MFAP2 and PTK7 was 1:200. All reagents, including blocked serum, secondary antibodies, and DAB colorimetric reagents, were purchased from Maixin Reagent (China).

### Cell/Tissue Immunofluorescence

Cell/Tissues were blocked with goat serum and incubated with the primary antibody overnight at 4 °C and subsequently incubated with secondary antibody conjugated with green or red dye for 45 min. Nucleus was stained using DAPI for 15 min. A fluorescence microscope (Nikon, Tokyo, Japan) was used to acquire images.

### Enzyme-linked immunosorbent assay (ELISA)

The level of extracellular MFAP2 was determined using an MFAP2 enzyme-linked immunosorbent assay (ELISA) kit (Mlbio, Shanghai, China). The supernatant was collected, and the extracellular MFAP2 level was measured following the manufacturer’s protocols.

### Virus and plasmid transfection

The silencing virus and overexpression plasmid of MFAP2 were purchased from Gikay Gene (China, Shanghai), whereas the overexpression virus was procured from GenePharma(China, Suzhou). The overexpression virus of PTK7 was obtained from Synbiotics, and the silencing virus was procured from GenePharma. The cells were digested and resuspended; 1 × 10^5^ cells were added to a 12-well plate with an appropriate amount of virus, and the fluid was changed after 24 hours. Finally, a stable transfected cell line was screened using 2 µg/mL of puromycin. The transfection reagent for plasmid transfection was Higene. The plasmid was mixed with Higene for 15 min and added to the culture medium. The medium was changed after 4 hours, and the cells were collected after 48 hours.

### Cell-cell adhesion and invasion assay

The cell–cell adhesion and invasion assay was performed as previously described [43, 44]. HPMCs were prepared for the co-culture of GC cells and laid flat on a 12-well plate for adhesion experiments. The GC cells were incubated in 5 μmol/L Calcein AM (Sigma-Aldrich) for 1 hours, digested with trypsin, and added to the prepared 12-well plate. The plate was washed with PBS to remove unattached GC cells. The cells were counted under a fluorescence microscope. For the cell-cell invasion experiment, two invasion models are involved. We applied mesothelial cells on top of matrix gel to simulate the process of gastric cancer cells invading the peritoneal mesothelial layer in vitro, in order to confirm that MFAP2 secreted by mesothelial cells promotes peritoneal metastasis of tumor cells. The matrix gel was added into the chamber (8-μm aperture; Corning), and then HPMCs co-cultured with GC cells were inoculated for 4–6 hours. Then, 5 × 10^5^ GC cells were added to the upper layer in 100 μL of serum-free medium. Next, 600 μL of culture medium containing 10% FBS was added to the lower layer. In addition, we placed mesothelial cells in the pore plate under the chamber and added matrix gel to the chamber (8-μm pore size; Corning. Then, add 5 × 10^5^ GC cells to the upper layer of 100 μL serum-free medium. Using the transwell indirect model to confirm that mesothelial cells enhance the invasive ability of cancer cells by secreting MFAP2. We removed the small chamber after 20 h for fixation and staining, and the cells were counted under a microscope.

### Luciferase activity assay

5 × 10^5^ HGC-27 cells were seeded in 12-well plates and transfected with TK, GFP-Smad4, pGL3-MFAP2 (WT, Del, and Mut), pGL3-PTK7, and reporter plasmids. The cells were collected using the dual luciferase reporter assay kit (Promega, USA) following the manufacturer’s protocol after 48 hours. Renilla and firefly luciferase activities were measured using a fluorometer.

### Immunoprecipitation

The cells were lysed in RIPA lysis buffer, and total protein in whole-cell extracts was used for immunoprecipitation (IP) with the indicated antibodies and protein A-Sepharose (GE Healthcare) at 4 °C for 12 hours. The immunoprecipitated proteins were separated via SDS-PAGE.

### Chromatin immunoprecipitation (ChIP)

For the Chromatin immunoprecipitation, an Pierce™ Magnetic ChIP Kit (Thermo Scientific) was used and obtained following the manufacturer’s instructions. Cells were cross-linked with 1% formaldehyde for 10 min and neutralized with 0.125 M glycine. Cells were disrupted by sonication, and protein-DNA complexes were precipitated with IgG or p-STAT3 antibodies (5 μg). qPCR was carried out with primers specific to the PTK7 promoter region.

### Cytoplasmic and nuclear protein extraction

Cytoplasmic and nuclear proteins were extracted using the Nuclear and Cytoplasmic Protein Extraction Kit (Beyotime, Shanghai, China).

### In vivo GCPM assay

The experiment used twelve 4-week female BALB/c nude mice randomly divided into two groups. Then, 2 × 10^6^ HGC-27 cells and 5 × 10^5^ control/shMFAP2 (primary) mesothelial cells were mixed in PBS at a ratio of 4:1 for intraperitoneal injection. After 4 weeks, anesthesia was performed, and the size and number of tumors in the abdominal cavity were compared. All experimental procedures were conducted in compliance with the Guidelines for the Care and Use of Laboratory Animals and approved by the Animal Experiment Ethics Committee of China Medical University.

### Statistical analysis

The experiments were carried out at least 3 times, and the representative results were shown. All experimental results were expressed as mean ± SEM. To compare the expression of targets between two groups, *p* values were calculated using two-tailed Student’s test. One-way ANOVA analysis was used to compare data between 3 groups. For the survival curves of patients between different groups, *p* values were calculated using log-rank testing. All statistical analyses used Prism 10.0 (GraphPad Software Inc., La Jolla, CA). A value of *p* < 0.05 was considered statistically significant and *p* value < 0.05 was marked with (^*^), < 0.01 with(^**^), < 0.001 with (^***^), or < 0.0001 with (^****^).

## Supplementary information

Supplementary data

The full uncropped Gels and Blots images

## Data Availability

The datasets generated and/or analyzed during the current study are not publicly available due to they are owned and managed by Cancer Hospital of Dalian University of Technology but are available from the corresponding author on reasonable request.
