# Supplementary material for: MFAP2 secreted by TGF-β1-induced cancer-associated fibroblast cells promotes gastric cancer peritoneal metastasis through Src-STAT3-PTK7 axis
Source: Cell Death Discov. 2026 Jul 8;12:393. doi: 10.1038/s41420-026-03243-3 (PMC13631357; doi:10.1038/s41420-026-03243-3)

Figure 1L：

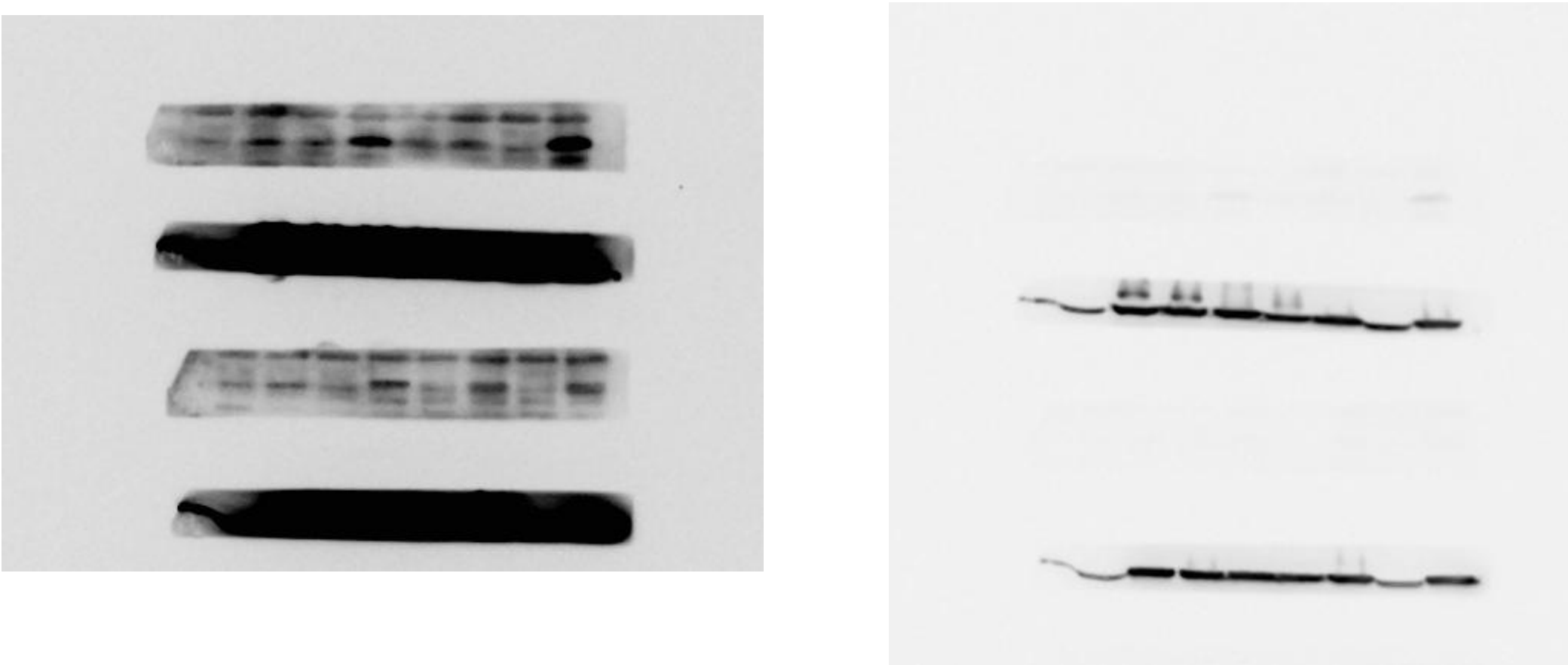

Figure2A

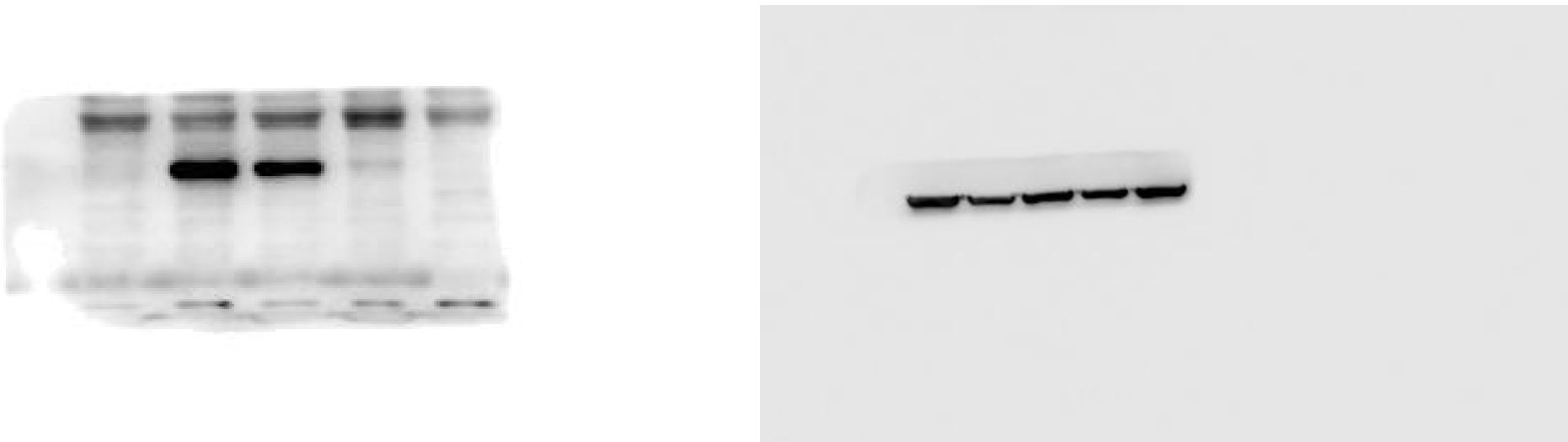

Figure2E

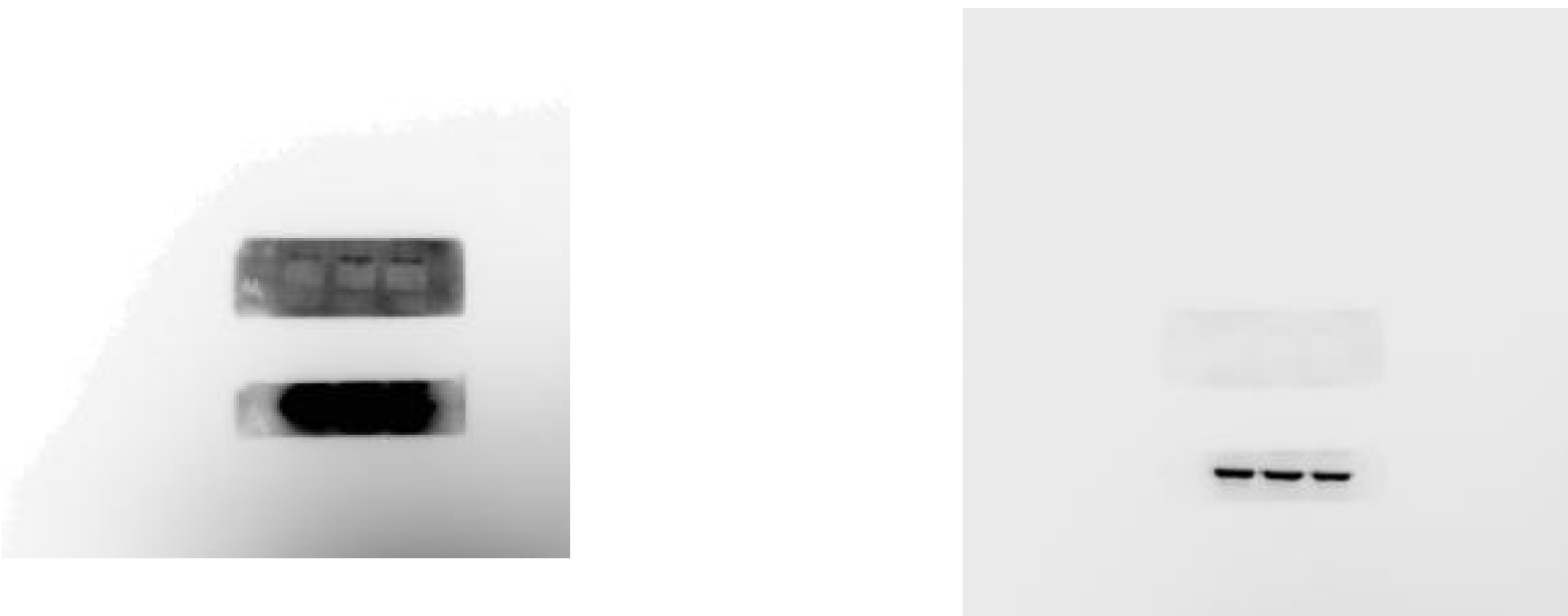

Figure3E

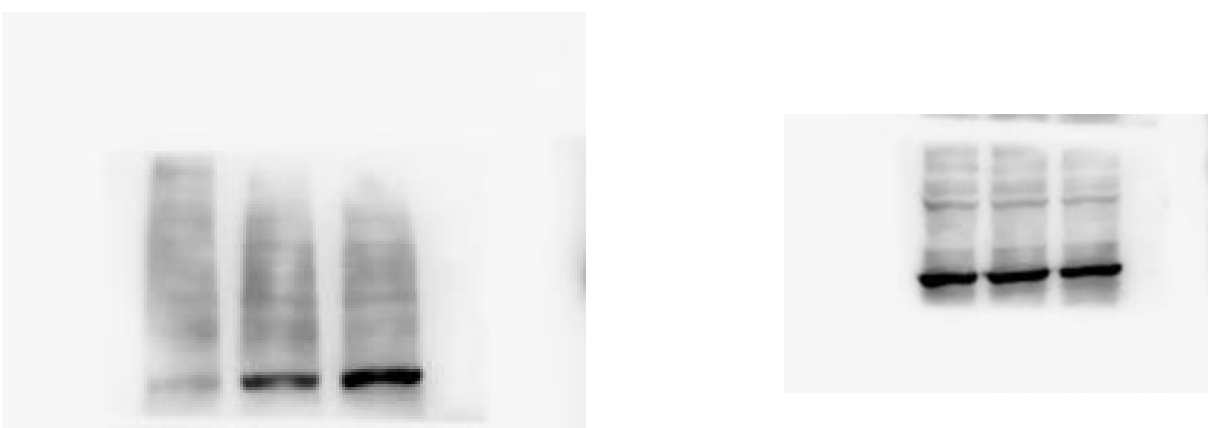

Figure3I

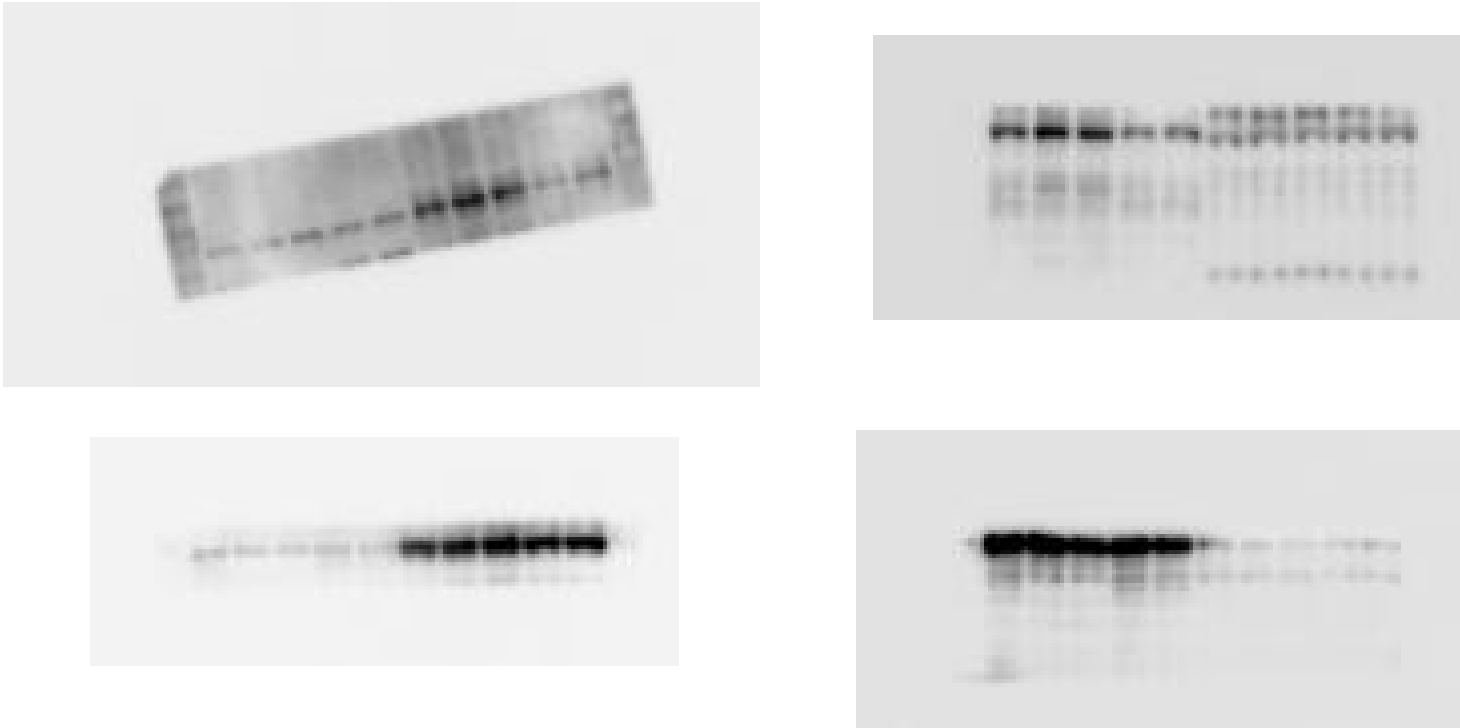

Figure5C

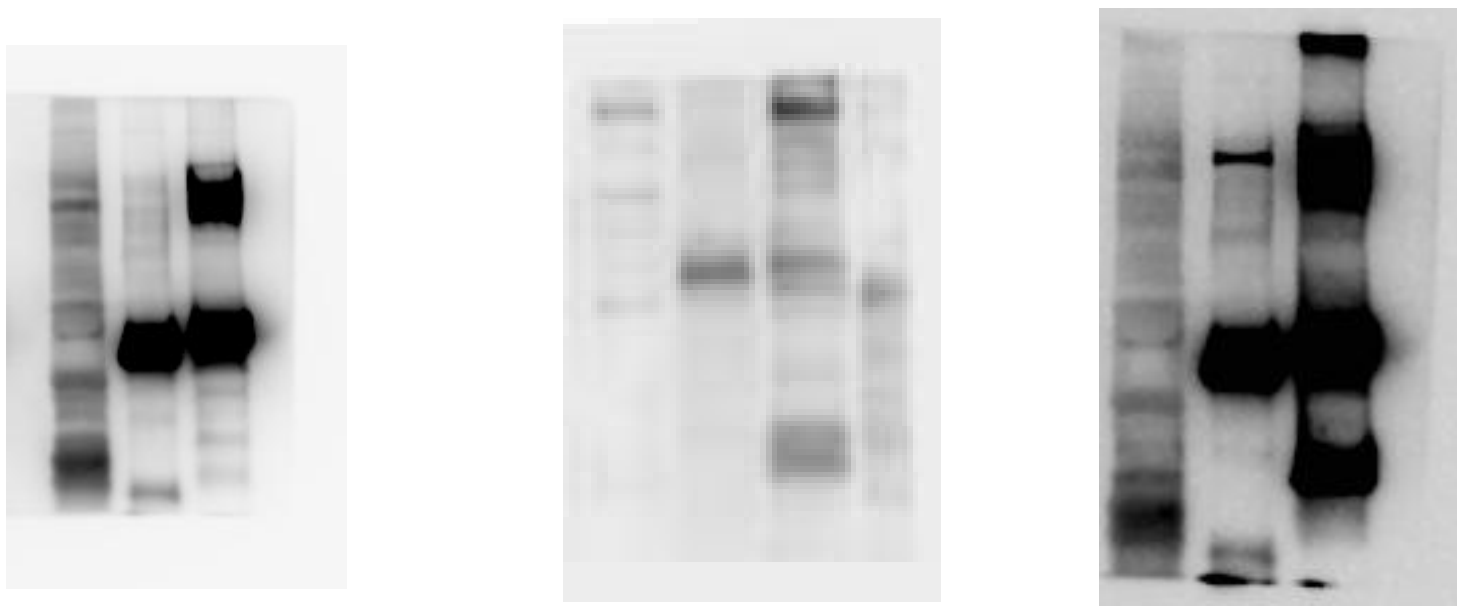

Figure6C

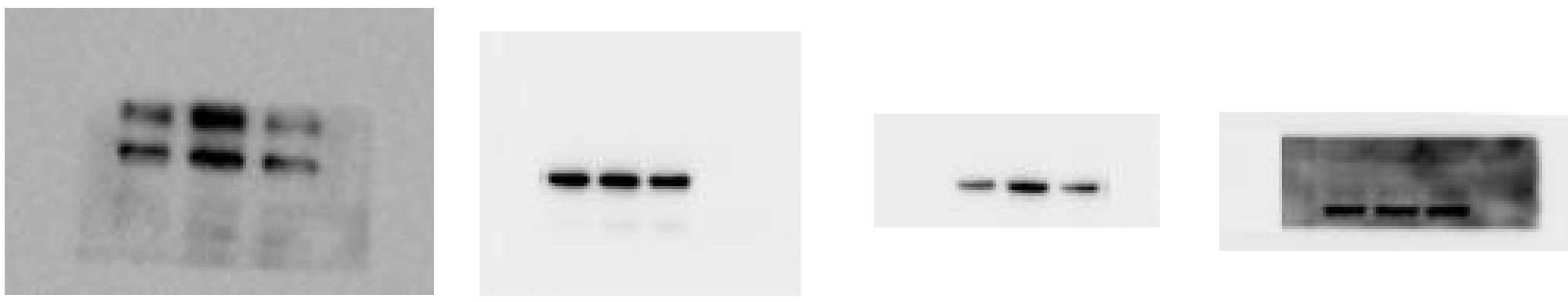

Figure6D

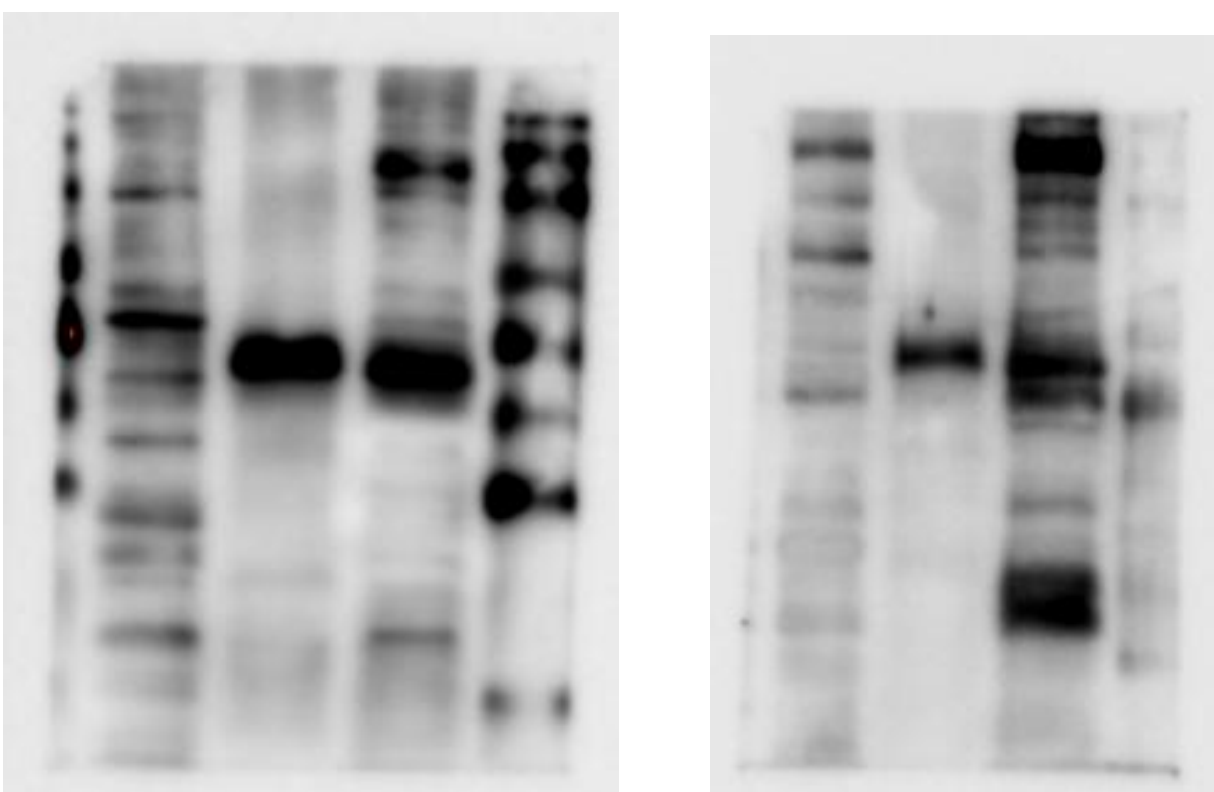

Figure6E

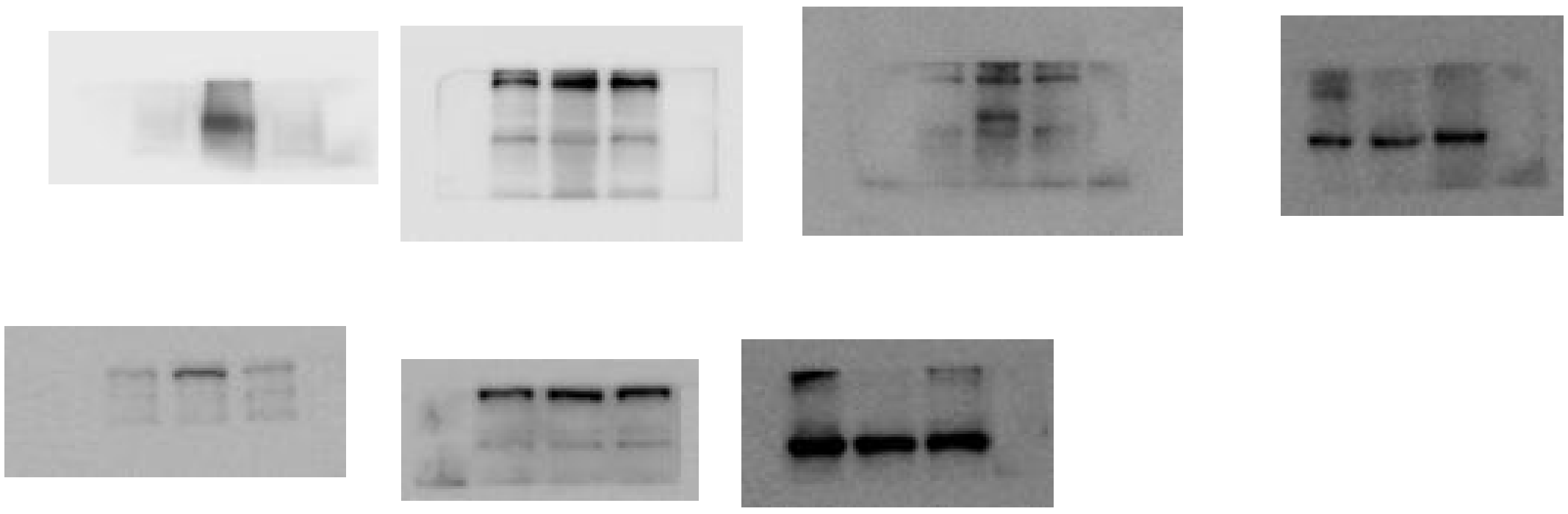

Figure6G

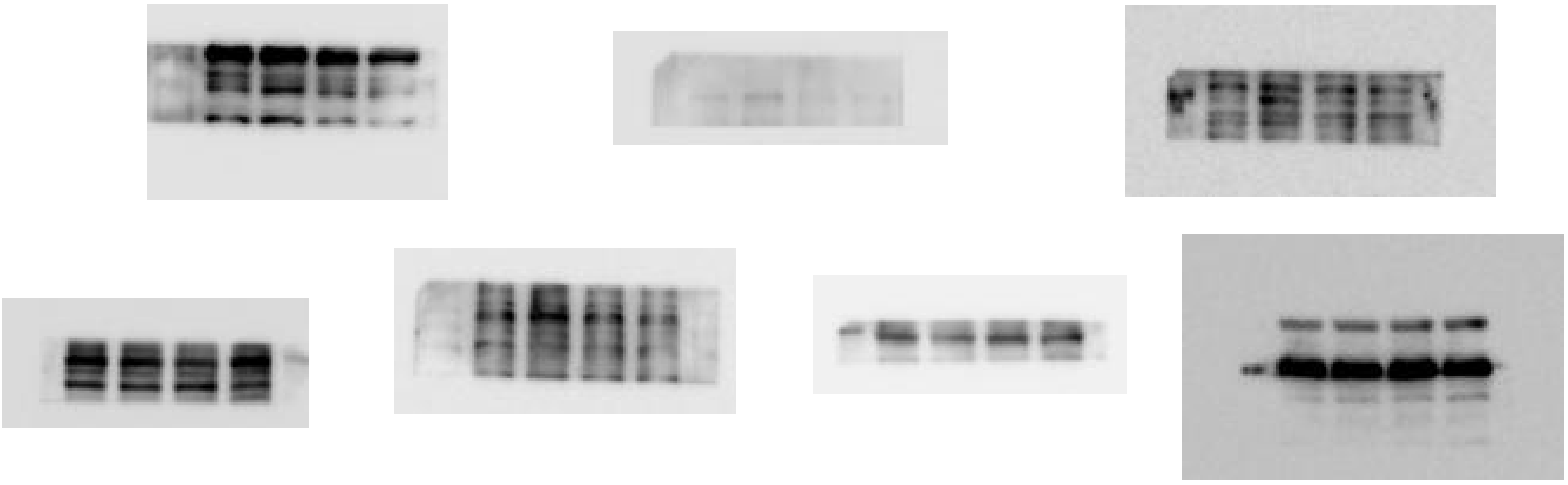

Figure6H

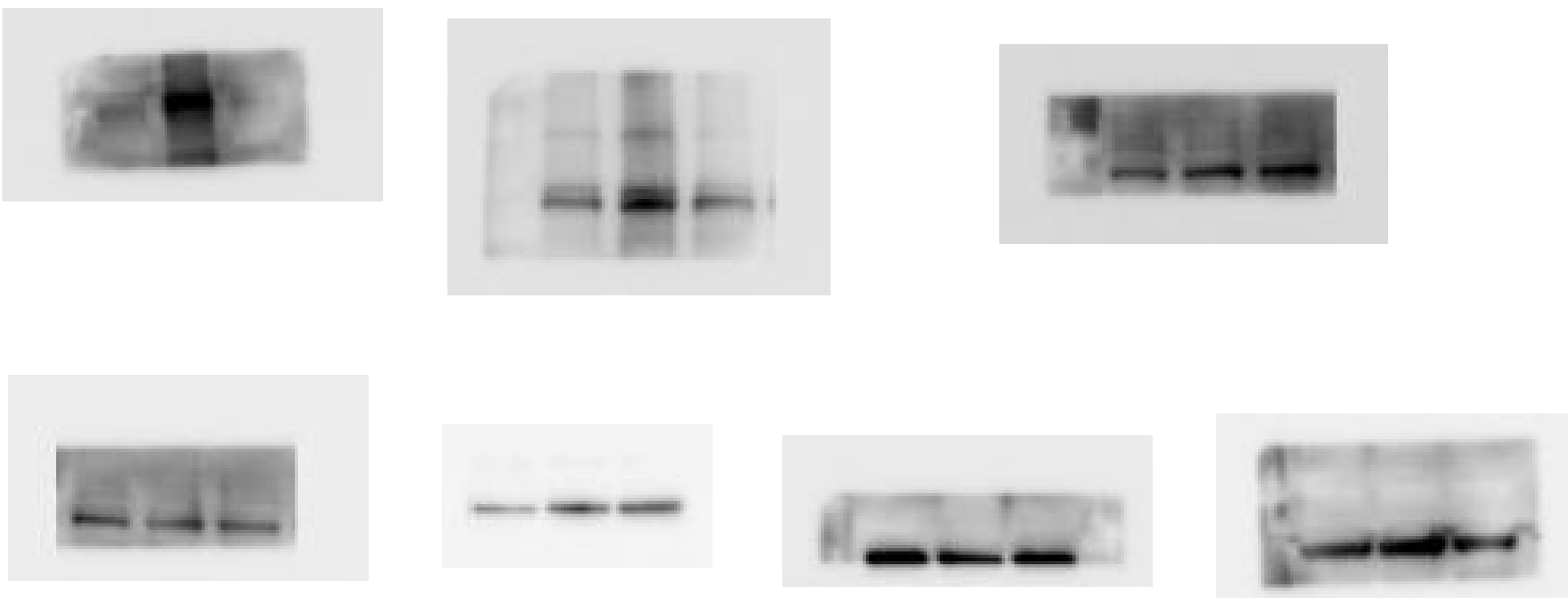

Figure6J

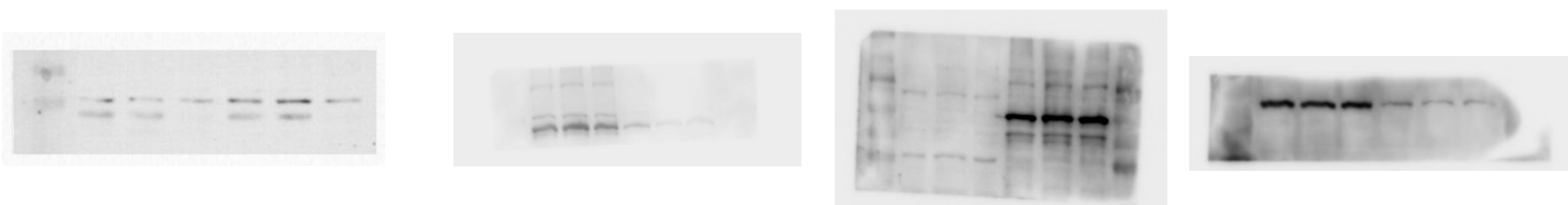

Figure6M

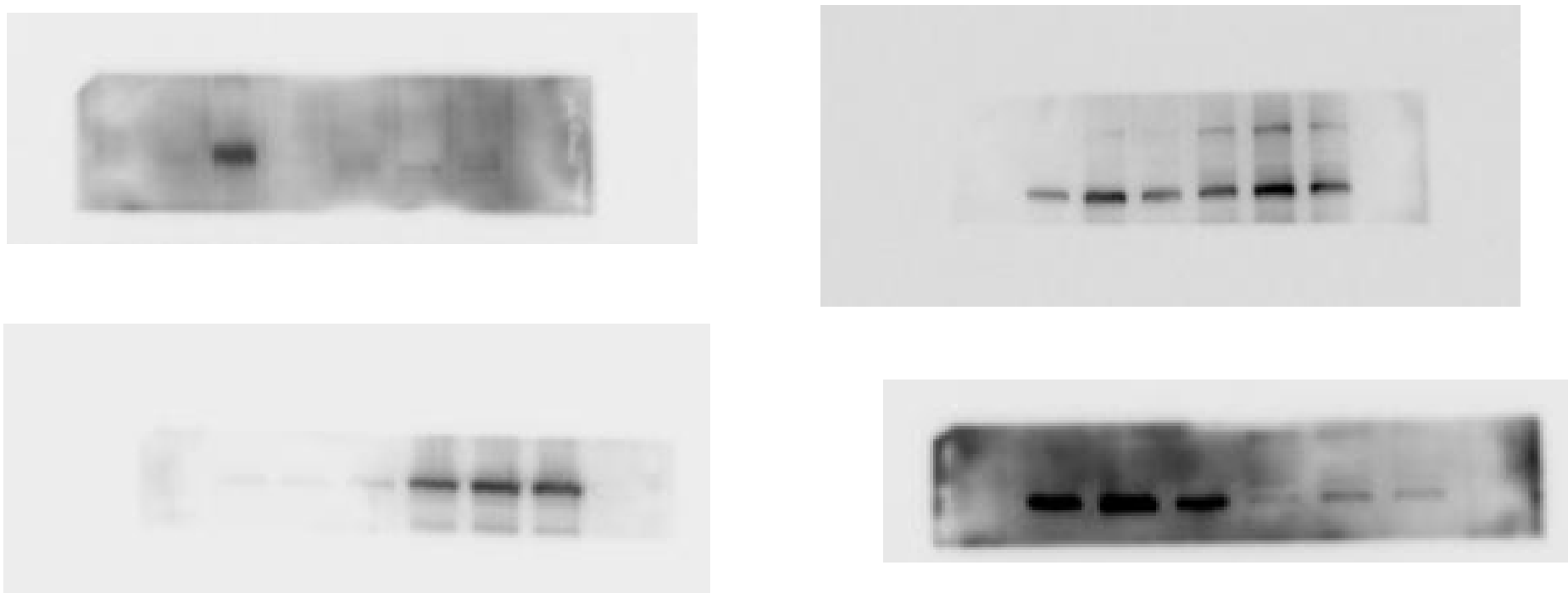

Figure6N

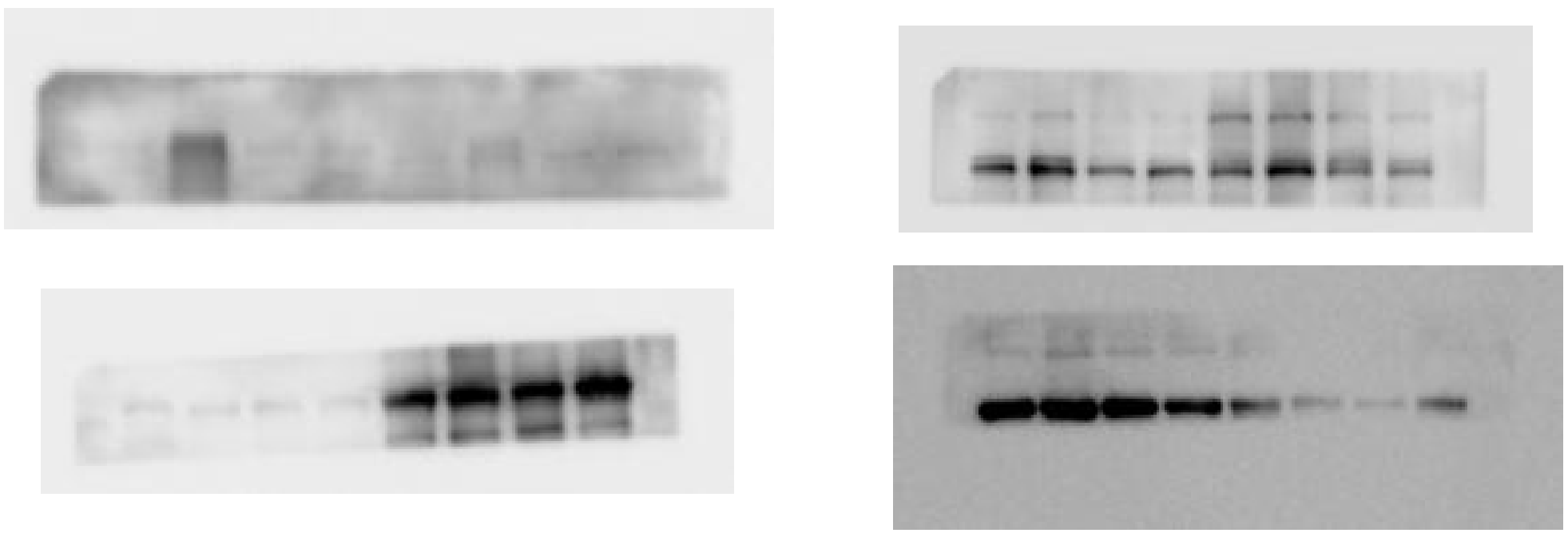

FigureS4E

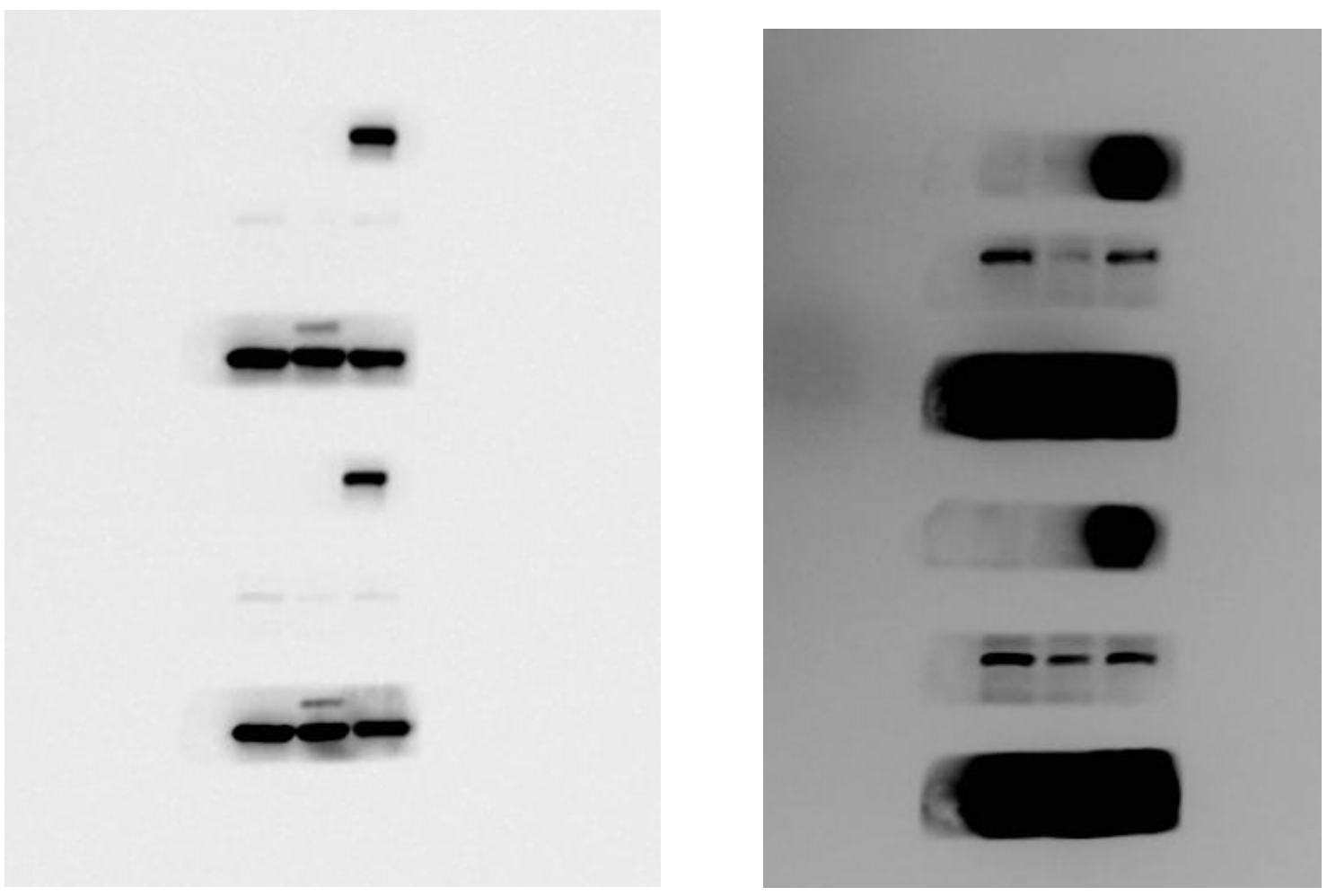

Supplement: Supplementary file 1 [file 41420_2026_3243_MOESM2_ESM.pdf]
