# Supplementary material for: MFAP2 secreted by TGF-β1-induced cancer-associated fibroblast cells promotes gastric cancer peritoneal metastasis through Src-STAT3-PTK7 axis
Source: Cell Death Discov. 2026 Jul 8;12:393. doi: 10.1038/s41420-026-03243-3 (PMC13631357; doi:10.1038/s41420-026-03243-3)
Supplement: Supplementary file 2 [file 41420_2026_3243_MOESM1_ESM.docx]

**Figure S1. MFAP2 expression positively correlated with poor prognosis of gastric cancer.**

(A) Representative images of IHC staining, with the box area in the top image magnified in the bottom image at a magnification of 100× the original magnification

(B) Expression of MFAP2 in adjacent and GC tissues. The relative staining of all samples are shown in the scatter plot (t test, ^****^*p* < 0.0001)

(C) Kaplan–Meier plot of OS in patients with cancer stratified by MFAP2 expression levels

(D) Representative phase contrast microscopy images of ascites cells 7 days (Day 7) and 20 days (Day 20) after culture are shown.

(E) Representative images of Immunofluorescence of cytokeratin and calretinin in the ascites cells after culture (day 7 and day 20) from a GC patient. Scale bar = 100 µm.

(F) Control or shMFAP2 primary mesothelial cells and tumor cells were injected into the abdominal cavity (5 mice per group). The red arrow in the representative image indicates the tumor

(G) Statistical chart of the number of tumor nodules (left) and the total weight of omental cancer nodules (right) in nude mice. ^****^*p* < 0.0001

(H) Hematoxylin and eosin staining of omental cancer nodules. On the right is the proportion of tumor cells in the metastatic nodule. ^***^*p* < 0.001

**Figure S2. TGF-β1 is associated with malignant progression, metastasis, and poor prognosis in cancer patients.**

(A) The Venn diagram displays the cross results of the top 150 upregulated DEGs (based on p-value ranking)

(B) Predict the exprssion of TGF-β1, TGFBR1 and TGFBR2 in different stages in TCGA database

(C) Predict the exprssion of TGF-β1, TGFBR1 and TGFBR2 in patients with or without metastatic gastric cancer in TCGA database

(D) Predict the overall survival of TGF-β1 in TCGA database

(E) GSEA enrichment analysis showed that MFAP2 was involved in the TGF-β1 pathway

(F) Analysis of the correlation between TGF-β1 and MFAP2 expression in GEO gastric cancer databases

**Figure S3. Network diagram of epithelial cells and eight clusters of fibroblasts.**

**Figure S4. PTK7 was the downstream factor of MFAP2/integrin αVβ3.**

(A) Heatmap analysis of differentially expressed genes regulated by MFAP2. C represents the control group, and T represents the group with the addition of MFAP2 purified protein

(B) GO analysis of differential gene enrichment pathways

(C) GSEA enrichment analysis of differentially expressed genes

(D) Analysis of the correlation between PTK7 and MFAP2 expression in GEO gastric cancer databases

(E) AGS (left) and HGC27 (right) cells transfected with the indicated Flag-PTK7 and co-cultured with Sv5 cells transfected with control or shMFAP2 for 48 h. Western blotting with the indicated antibodies

(F) Conducted quantitative analysis on the expression of phosphorylated proteins (p-Src, p-STAT3) and total proteins (total Src, total STAT3). The intensity of phosphorylated protein bands in Figure 6E was normalized to total protein intensity, ^****^*p* < 0.0001

(G) Conducted quantitative analysis on the expression of phosphorylated proteins (p-Src, p-STAT3) and total proteins (total Src, total STAT3). The intensity of phosphorylated protein bands in Figure 6G was normalized to total protein intensity, ^****^*p* < 0.0001

(H) Conducted quantitative analysis on the expression of phosphorylated proteins (p-Src, p-STAT3) and total proteins (total Src, total STAT3). The intensity of phosphorylated protein bands in Figure 6H was normalized to total protein intensity, ^****^*p* < 0.0001

(I)Jasper predicted the binding sequence of STAT3 and PTK7

(J)Transfection of control or shMFAP2 into mesothelial cells and control or PTK into GC cells, co-cultured for 48 h, followed by adhesion/invasion analysis. Statistical histogram of cell adhesion/invasion analysis in (D) shows that the results of three independent experiments are representative (^**^*p* < 0.01, ^***^*p* < 0.001, ^****^*p* < 0.0001)

**Table S1. Correlation between MFAP2 expression and clinicopathologic feathers in GC patients.**

|  | **MFAP2 expression** | |  |
| --- | --- | --- | --- |
| **Factor** | **Low (n=122)** | **High (n=85)** | **P value** |
| Sex |  |  | 0.910 |
| Male | 87 | 60 |  |
| Female | 35 | 25 |  |
| Age |  |  | 0.475 |
| ≤60 | 65 | 41 |  |
| ＞60 | 57 | 44 |  |
| Tumor location |  |  | 0.291 |
| Upper | 19 | 8 |  |
| Middle | 21 | 20 |  |
| Lower | 82 | 57 |  |
| Tumor size |  |  | **0.001** |
| ≤4 | 38 | 7 |  |
| ＞4 | 84 | 78 |  |
| Histological type |  |  | 0.393 |
| Well/moderate | 59 | 36 |  |
| Poor | 63 | 49 |  |
| Borrman type |  |  | **0.001** |
| Ⅰ-Ⅲ | 107 | 54 |  |
| Ⅳ | 15 | 31 |  |
| T stage |  |  | **0.045** |
| T1-T2 | 24 | 8 |  |
| T3-T4 | 98 | 77 |  |
| TNM stage |  |  | **0.003** |
| Ⅰ,Ⅱ | 50 | 18 |  |
| Ⅲ, Ⅳ | 72 | 67 |  |
